# Quantifying device type and handedness biases in a remote Parkinson’s disease AI-powered assessment

**DOI:** 10.1038/s41746-025-01934-2

**Published:** 2025-08-27

**Authors:** Zerin Nasrin Tumpa, Md Rahat Shahriar Zawad, Lydia Sollis, Shubham Parab, Irene Y. Chen, Peter Washington

**Affiliations:** 1 University of Hawaii at Manoa, Honolulu, HI USA; 2 New York University, New York, NY USA; 3 University of California Berkeley, Berkeley, CA USA; 4 University of California San Francisco, San Francisco, CA USA

**Keywords:** Diagnostic markers, Translational research, Information technology

## Abstract

We investigate issues pertaining to algorithmic fairness and digital health equity within the context of using machine learning to predict Parkinson’s Disease (PD) with data recorded from structured assessments of finger and hand movements. We evaluate the impact of demographic bias and bias related to device type and handedness. We collected data from 251 participants (99 with PD or suspected PD, 152 without PD or any suspicion of PD). Using a random forest model, we observe 92% accuracy, 94% AUROC, 86% sensitivity, 92% specificity, and 84% F1-score. When examining only F1-score differences across groups, no significant bias appears. However, a closer look reveals bias regarding positive prediction and error rates. While we find that sex and ethnicity have no statistically significant impact on PD predictions, biases exist regarding device type and dominant hand, as evidenced by disparate impact and equalized odds. Our findings suggest that remote digital health diagnostics may exhibit underrecognized biases related to handedness and device characteristics, the latter of which can act as a proxy for socioeconomic factors.

## Introduction

PD is the fastest-growing neurological disorder in the world^1^. It is projected that the number of individuals aged 50 years or older with PD in the USA will rise by 128% from about 340,000 in 2005 to around 610,000 by 2030^2^. Numerous studies have emphasized the possibility of diagnosing PD through motor patterns such as verbal fluency^3^, voice signals-voice disorders^4,5^, speech^6^, handwritten trace^7^, and gait patterns^8^. Finger tapping is one of the most reliable tests for motor performance^9^. The necessity for lab equipment and specialist supervision has made the diagnostic process complex and expensive^10^.

We developed a web-based remote digital health tool that operates through a web-based interface to predict whether a user has PD or symptoms that would lead them to suspect a diagnosis of PD. The system captures motor function data through structured interaction tasks involving mouse tracing and keyboard pressing as well as memory tests conducted through a user-friendly platform accessible on standard laptop and desktop computers. Using these data, we trained machine learning models to classify individuals into categories of PD or Non-PD. Our pilot study^11^ explored the feasibility of an earlier version of our web application, which demonstrated the potential for remote PD disease screening using digital motor assessments. Our current study builds upon these findings by refining the data collection framework, expanding participant recruitment from a small sample to over 250 participants, and, in particular, systematically evaluating bias with respect to several factors, including understudied aspects in remote digital health assessments: device type and handedness.

We collected data from a single assessment lasting less than 15 min of participants who engaged in structured interactive tasks on a remotely accessible web application that we created. Each assessment consisted of a series of mouse tracing and keyboard pressing tasks to measure motor movement. For the key press tasks, metrics such as response time, accuracy, and accidental key presses from unintended movements were recorded. For the mouse trace tasks, where participants were asked to trace straight and curved paths displayed on-screen using a trackpad or mouse, we measured the stability and precision of two-dimensional hand movements, providing insights into motor control and coordination of the lower arm.

Although this study describes a remote PD assessment tool, the primary contribution of this work is with respect to the issue of algorithmic fairness, defined as the principle that machine learning systems should yield equitable performance across diverse demographics and socioeconomic proxies. While prior studies have explored remote digital health assessments for PD^12–17^, our study delves into issues of algorithmic fairness in remote PD assessments in particular and consumer digital health informatics more broadly by exploring the performance of machine learning models across diverse demographic groups as well as understudied attributes like handedness and device type.

Studying such issues is crucial for the field of consumer digital health, which aims to make healthcare more accessible. Underrepresentation of specific subgroups in the training data may result in reduced model generalizability and diminished performance for those populations. For instance, if left-handed individuals or non-white participants are underrepresented in the data, the resulting models may exhibit disproportionately lower predictive performance for those groups. Similarly, technical heterogeneity across input devices, such as differences in cursor resolution, sampling rates, input latency, and sensitivity between operating systems (e.g., Windows vs. macOS) can introduce variability in the motor data captured. These device-level variations may inadvertently correlate with demographic factors, including socioeconomic status, thereby embedding confounding patterns into the data. Such biases create a paradox in remote digital health assessments by exacerbating health disparities in a tool that was designed to reduce disparities.

By examining factors such as sex (male, female), race (White, Non-White), dominant hand (left, right), and device type (Windows, Mac) using multiple fairness metrics, we aim to understand and highlight potential biases in the predictions made by a remote digital PD assessment. We note in particular that exploring algorithmic fairness with respect to device type and dominant hand remains an understudied aspect of consumer digital health informatics.

## Results

### Novel remotely curated dataset

**Fig. 1:**
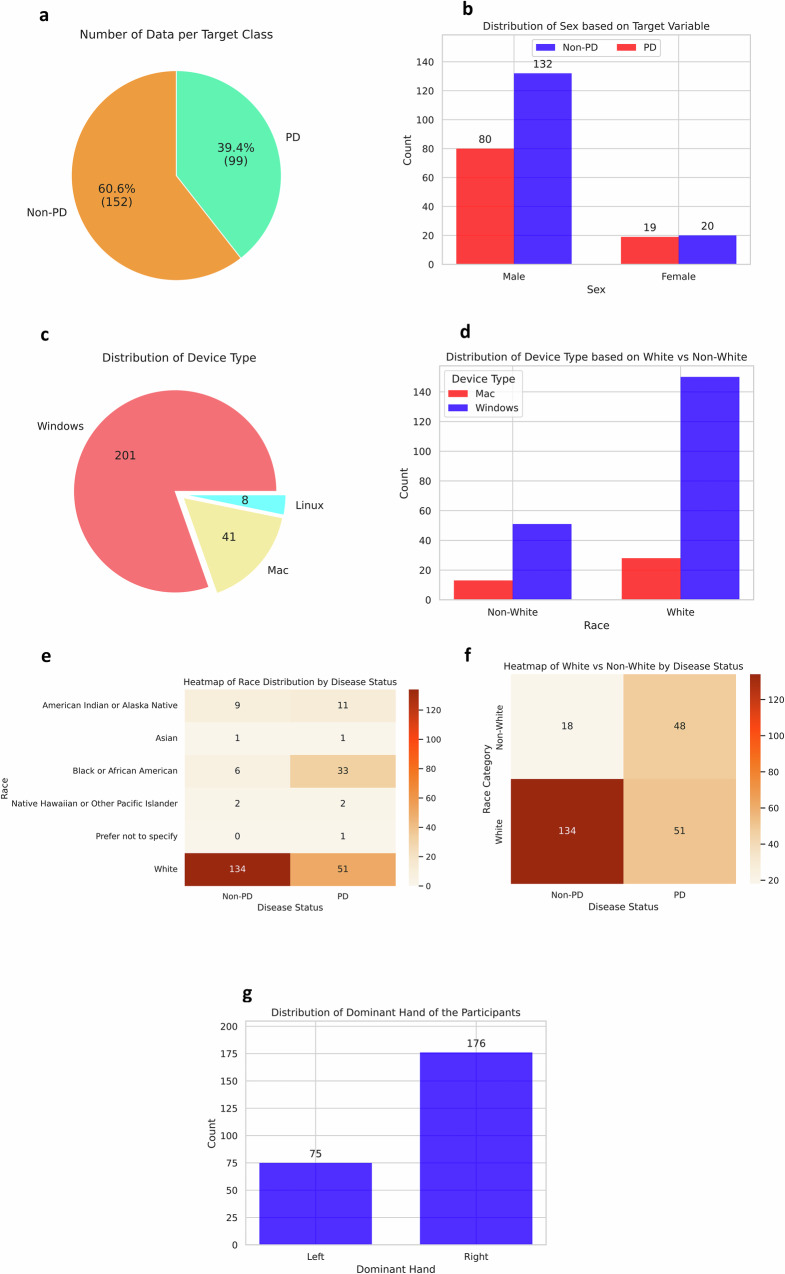
Data distributions before any upsampling. **a** The dataset is class imbalanced, with ~39.4% PD patients and 60.6% Non-PD participants. **b** The total number of male participants is much higher than that of female participants, with differing class balances across sex groups. **c** Most of the participants used a Windows device. Two hundred and one participants used Windows, whereas only 41 users used a Mac, and eight used Linux. **d** Within the White group, Windows is the predominant device type, with 150 users, compared to only 28 users on Mac. Similarly, in the Non-White group, Windows usage remains higher, with 45 users, while Mac usage is much lower, at 12 users. This pattern indicates a strong preference for Windows devices across racial groups, although the White group has a significantly larger sample size overall. **e** Of participants with PD, 51 are White, followed by Black or African American, with 33 responses. White participants consist of 134 responses in the Non-PD group, with only six responses from Black or African American participants. **f** Distribution of race by disease status, specifically comparing White and Non-White groups. **g** A majority of the participants reported their dominant hand as right (176), whereas only 75 participants identified as left-handed.

We recruited 251 individuals, providing a comprehensive dataset to analyze the effectiveness of our remotely accessible web-based application for detecting PD. Of these participants, 99 either self-reported a diagnosis of PD (73 participants) or self-reported as suspecting PD (26 participants). We merge these into a single diagnostic category of “PD” for the remainder of this paper. We also recruited 152 non-PD controls (Fig. 1a). We note that while we collected diagnoses through self report and combined PD and self-reported PD into one diagnostic category, the purpose of this paper is not to create a clinical-grade PD diagnostic tool but rather to evaluate biases in remote digital health assessment tools.

There was significant underrepresentation of many groups in our dataset, and we sought to study the effect of this imbalance on disparities in performance across groups. The sex distribution included 39 females and 212 males (Fig. 1b). Participants utilized various devices to access the web-based application: 201 used Windows desktops, 41 used Mac desktops, and 8 used Linux desktops (Fig. 1c). The device type distribution was roughly similar between White and Non-White groups (Fig. 1d). The heatmap in Fig. 1e shows how individuals are distributed across different racial categories based on their PD status. The majority of participants were White (185). There were 39 Black or African American participants. Smaller groups included 20 American Indian or Alaska Native individuals, 4 Native Hawaiian or other Pacific Islander individuals, and 2 Asian participants (Fig. 1e). While the distribution of White and Non-White groups was balanced within the PD group, the Non-PD group was heavily skewed towards White participants (Fig. 1f). Most of the participants were right-handed, with 176 individuals identifying as right-handed and 75 as left-handed (Fig. 1g).

The remotely accessible application included seven structured tasks: three involving mouse exercises, three involving keyboard interactions, and one testing working memory. Each task consisted of three levels of increasing difficulty. The mouse-based tasks involved tracing straight lines, sine waves, and spirals, while the keyboard-based tasks included single-letter presses, multiple-letter presses, and randomized sequences, forming a comprehensive set of activities designed to test different motor and cognitive skills. The working memory game assessed cognitive function. From these interactions, we extracted 79 features across five major categories.

Similarly, in the Non-White group, Windows usage remains higher, with 45 users, while Mac usage is much lower, at 12 users. This pattern indicates a higher prevalence of Windows devices across racial groups. (e) Of participants with PD, 51 are White, followed by Black or African American, with 33 responses. White participants consist of 134 responses in the Non-PD group, with only six responses from Black or African American participants. (f) Distribution of race by disease status, specifically comparing White and Non-White groups. (g) A majority of the participants reported their dominant hand as right (176), whereas only 75 participants identified as left-handed.

### Model selection

**Fig. 2:**
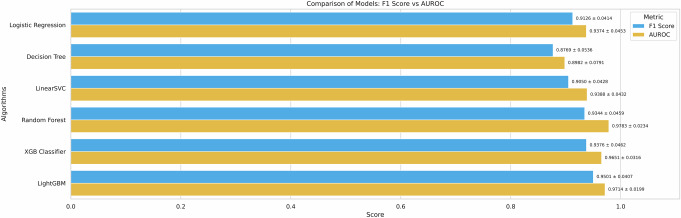
Comparison of classical machine learning models. The results are generated by performing cross-validation on our training data only. We report the mean with standard deviation as error bars across all the folds.

We first sought to select high-performing models for subsequent analyses. Using manually engineered features, we tested six classical machine-learning models for predicting PD. Each model was evaluated using 5-fold cross-validation on the training set, and the performance of the models was recorded using the mean F1-score and AUROC score across 5-folds (Fig. 2). The dataset was split into 70% training and 30% testing data before running cross-validation on the training data.

**Fig. 3:**
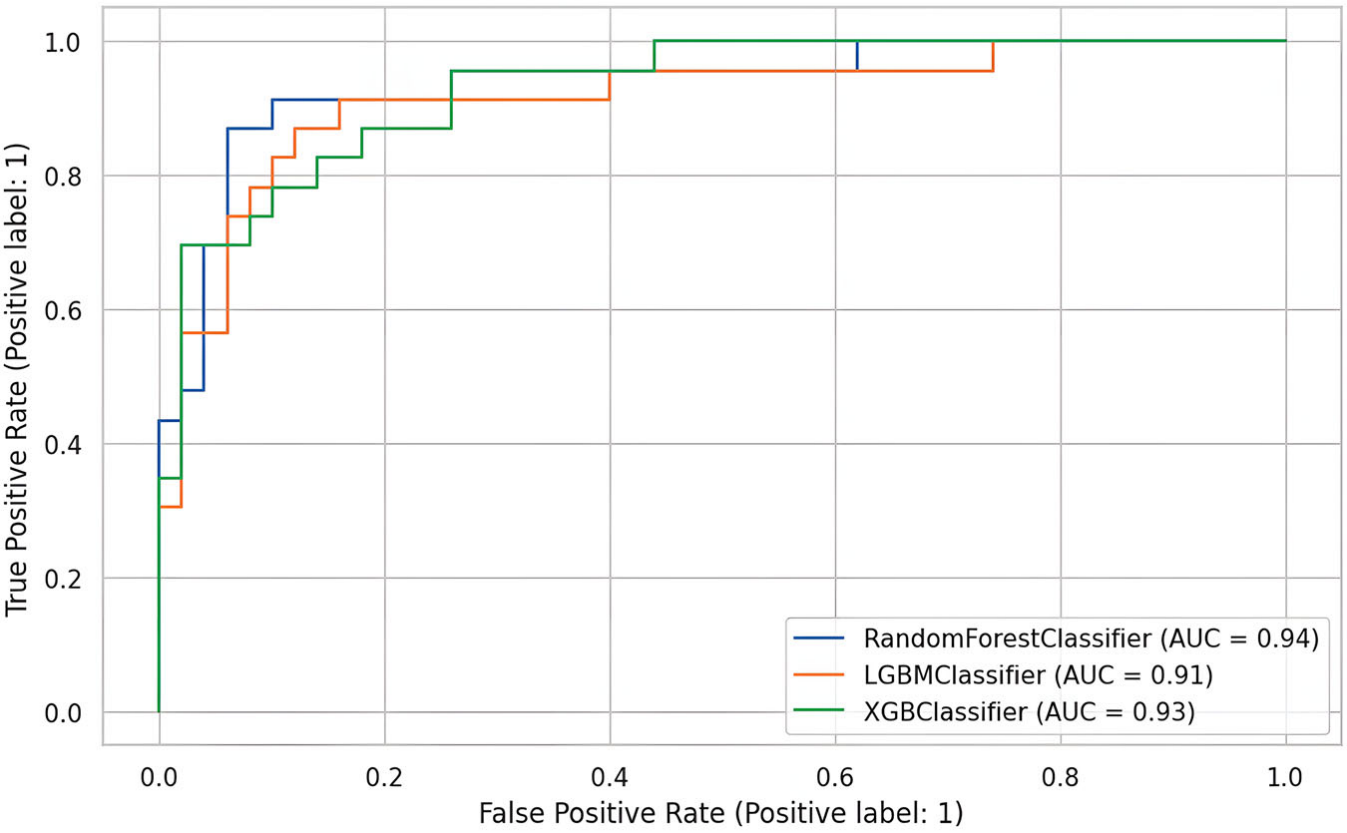
ROC curves and corresponding AUC scores for the random forest, LightGBM, and XGBoost classifiers. During the training phase, the three top-performing models were further tested on the held-out data to report their ROC curves and AUROC scores. The performance differences are minimal when comparing the ROC curves and the area under the curve (AUC). The Random Forest model achieved the highest AUC score of 94%, followed by XGBoost at 93% and LightGBM at 91%.

Although all the experimental models exhibited promising performance during this model selection stage, the LightGBM classifier outperformed the others by a small margin, achieving a 95.01% F1-score (4.07% standard deviation) and a 97.14% AUROC (1.99% standard deviation). The Random Forest, XGBoost, and LightGBM classifiers were selected to test their performance further on held-out test data. The 30% test data were used to make predictions using the three trained models (Table 1, Fig. 3).

**Table 1:** Performance of the top-performing ML models on held-out test data

	Accuracy	F1-score	AUROC	Sensitivity	Specificity
**Random forest**	92%	84%	94%	86%	92%
**LightGBM**	86%	78%	91%	78%	90%
**XGBoost**	86%	77%	93%	81%	88%

### Model bias and fairness

To evaluate bias, we used traditional accuracy metrics like F1-score (the harmonic mean of precision and recall), sensitivity, specificity, and AUC as well as fairness-specific metrics such as disparate impact (DI), equal opportunity (EO), and equalized odds (EOdds). For DI and EO, which are measured as a ratio across groups, a value of 1 indicates perfect fairness, with equal treatment of the privileged and unprivileged groups. Values less than 1 signify biases against the unprivileged group, while values greater than 1 indicate potential biases favoring the unprivileged group. EOdds measures differences in true and false positive rates; a value of 0 represents ideal fairness, with deviations indicating significant bias.

We selected male, White, Mac, and right-handed individuals as the privileged groups in the study (Table 2). While the selection of unprivileged groups corresponded to underrepresentation in our dataset in most cases, we chose to set Mac users as the privileged group despite underrepresentation due to the homogeneity of Mac devices—a contrast to Windows devices created by a wide range of manufacturers.

**Table 2:** Our selection of “Privileged” and “Unprivileged” groups in terms of the calculations for common algorithmic fairness metrics

Category	Unprivileged	Privileged
Sex	Female	Male
Race	Non-White (Black, Asian, Native Hawaiian, etc.)	White
Device	Windows	Mac
Dominant hand	Left	Right

**Fig. 4:**
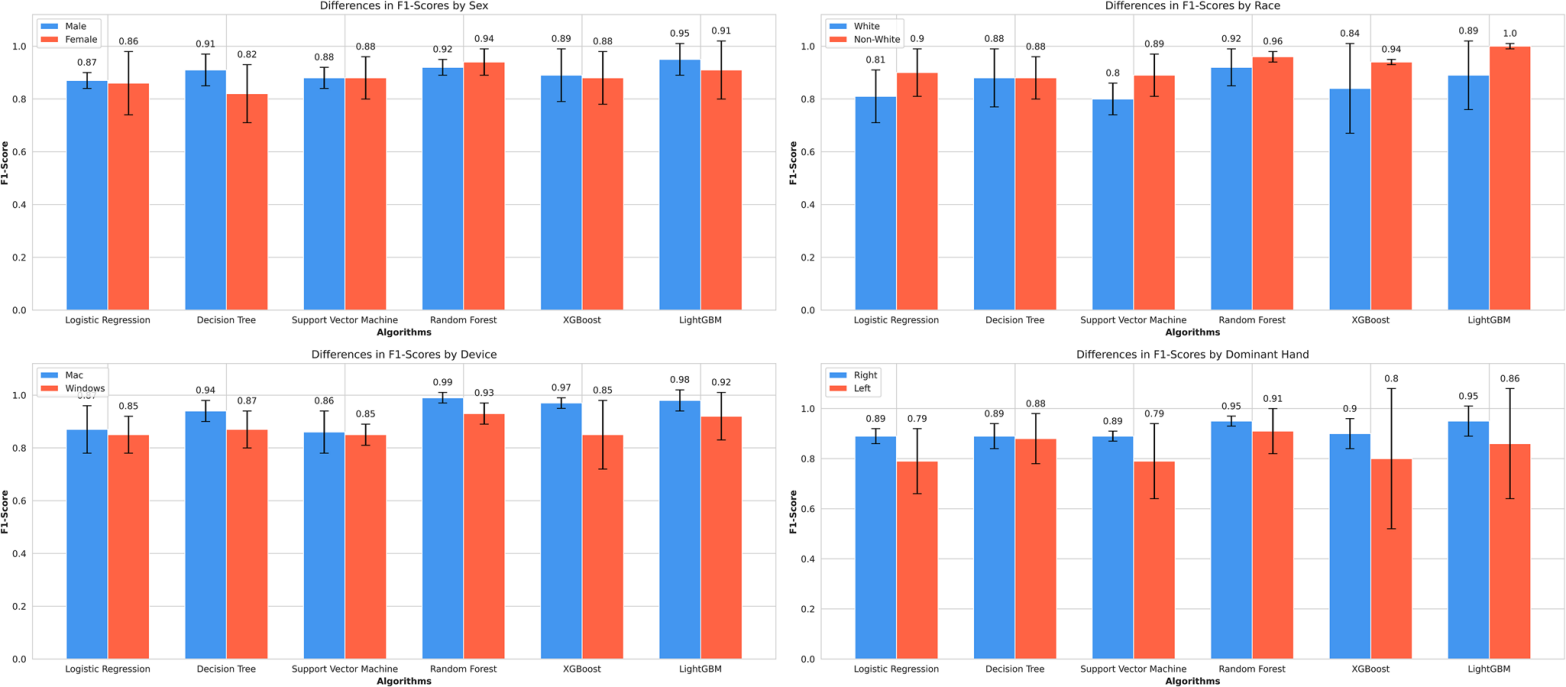
F1-score comparisons between the privileged and unprivileged groups for all protected attributes after race upsampling. No significant differences exist between the two groups in any category after upsampling by race.

Table 3 displays the F1-score, sensitivity, specificity, precision, and AUROC differences between groups for four attributes: sex, race, device type, and dominant hand. Table 3 shows these metrics following upsampling by race. Detailed performance metrics for the remaining three models are provided in Supplementary Table 1, while Supplementary Table 2 provides the values before any resampling was applied. Before upsampling, the Non-White group exhibited higher F1-scores than the White group, which was counterintuitive given the underrepresentation of Non-White data. Closer examination revealed that the “Non-White Non-PD” group had substantially less data than the “White Non-PD” group, likely resulting in an inflated F1-score for the Non-White group overall. Figure 1f illustrates the distribution of participants by race and disease status, clearly highlighting the underrepresentation of the “Non-White Non-PD” group. This is a salient example of how the underrepresentation of certain groups in machine-learning datasets can mask underlying biases. To address this imbalance, we increased the data in the “Non-White Non-PD” group to bring it closer in size to the White group using resampling with replacement. After race resampling, we observed that the F1-score gap between White and Non-White groups significantly narrowed, with no significant differences between the groups after upsampling (Supplementary Table 3). All sensitive categories no longer yielded statistically significant differences in F1-scores (Fig. 4). We also report group-wise binary indicators of statistically significant differences in sensitivity, specificity, and precision (Supplementary Tables 4, 5, and 6). These analyses, conducted after race upsampling, further support the observed bias patterns. Supplementary Tables 7, 8, 9, and 10 record the group-wise binary indicators of statistically significant differences in F1-score, sensitivity, specificity, and precision before race upsampling.

**Table 3:** Top three model performance for after race upsampling on each group-based sex, race, device type, and dominant hand

	F1-score		Sensitivity		Specificity		Precision		AUROC	
Sex	Male	Female	Male	Female	Male	Female	Male	Female	Male	Female
Random forest	0.95 (0.03)	0.92 (0.06)	0.98 (0.03)	1.00 (0.00)	0.93 (0.03)	0.75 (0.28)	0.93 (0.04)	0.87 (0.11)	0.99 (0.02)	0.94 (0.10)
XGBoost	0.89 (0.10)	0.88 (0.10)	0.94 (0.05)	0.95 (0.06)	0.83 (0.23)	0.75 (0.25)	0.86 (0.15)	0.84 (0.14)	0.97 (0.02)	0.92 (0.11)
LightGBM	0.95 (0.06)	0.91 (0.11)	0.98 (0.02)	1.00 (0.00)	0.92 (0.12)	0.77 (0.25)	0.92 (0.11)	0.85 (0.17)	0.99 (0.01)	0.97 (0.03)
**Race**	**White**	**Non-White**	**White**	**Non-White**	**White**	**Non-White**	**White**	**Non-White**	**White**	**Non-White**
Random forest	0.92 (0.07)	0.97 (0.02)	0.96 (0.03)	0.99 (0.02)	0.53 (0.43)	0.58 (0.47)	0.88 (0.10)	0.95 (0.04)	0.96 (0.05)	1.00 (0.00)
XGBoost	0.84 (0.17)	0.94 (0.01)	0.94 (0.05)	0.93 (0.06)	0.39 (0.42)	0.58 (0.47)	0.80 (0.23)	0.95 (0.05)	0.95 (0.03)	1.00 (0.00)
LightGBM	0.89 (0.14)	1.00 (0.01)	0.96 (0.03)	0.99 (0.02)	0.46 (0.41)	0.60 (0.49)	0.84 (0.19)	1.00 (0.00)	0.95 (0.02)	1.00 (0.00)
**Device**	**Mac**	**Windows**	**Mac**	**Windows**	**Mac**	**Windows**	**Mac**	**Windows**	**Mac**	**Windows**
Random forest	0.99 (0.02)	0.92 (0.05)	1.00 (0.00)	0.97 (0.03)	0.82 (0.20)	0.92 (0.05)	0.98 (0.03)	0.89 (0.06)	1.00 (0.00)	0.98 (0.04)
XGBoost	0.97 (0.02)	0.85 (0.12)	0.98 (0.04)	0.91 (0.07)	0.79 (0.26)	0.84 (0.18)	0.97 (0.04)	0.81 (0.17)	1.00 (0.00)	0.97 (0.03)
LightGBM	0.98 (0.04)	0.92 (0.09)	1.00 (0.00)	0.97 (0.03)	0.80 (0.40)	0.91 (0.11)	0.97 (0.07)	0.89 (0.14)	1.00 (0.00)	0.98 (0.02)
**Dominant Hand**	**Right**	**Left**	**Right**	**Left**	**Right**	**Left**	**Right**	**Left**	**Right**	**Left**
Random forest	0.95 (0.03)	0.88 (0.10)	0.98 (0.02)	0.94 (0.05)	0.90 (0.07)	0.96 (0.04)	0.92 (0.04)	0.85 (0.13)	0.99 (0.02)	0.96 (0.07)
XGBoost	0.91 (0.07)	0.79 (0.28)	0.94 (0.05)	0.96 (0.08)	0.84 (0.15)	0.84 (0.25)	0.88 (0.10)	0.76 (0.32)	0.97 (0.02)	0.98 (0.03)
LightGBM	0.95 (0.06)	0.86 (0.21)	0.98 (0.02)	0.98 (0.03)	0.90 (0.13)	0.92 (0.12)	0.93 (0.09)	0.82 (0.27)	0.99 (0.01)	0.99 (0.02)

Most algorithms achieved DI scores somewhat close to 1 for sex and race after race resampling (Table 4). However, due to consistently high standard deviations, we cannot make any conclusive statements about whether sex or race significantly influences the models’ distribution of predictions towards any particular group.

**Table 4:** Bias metrics of the top three models based on sex, race, device type, and dominant hand from the cross-validation with bootstrap sampling after race upsampling

Sex			
	Disparate impact	Equal opportunity	Equalized odds
**Random forest**	1.07 (0.59)	1.02 (0.03)	0.23 (0.22)
**XGBoost**	1.03 (0.59)	1.02 (0.03)	0.28 (0.21)
**LightGBM**	1.09 (0.55)	1.02 (0.03)	0.17 (0.21)
**Race**			
**Random forest**	1.41 (1.14)	1.03 (0.03)	0.09 (0.05)
**XGBoost**	1.24 (1.14)	0.99 (0.01)	0.22 (0.29)
**LightGBM**	1.44 (1.43)	1.03 (0.03)	0.15 (0.20)
**Device**			
**Random forest**	0.48 (0.05)	0.96 (0.03)	0.15 (0.14)
**XGBoost**	0.58 (0.14)	0.94 (0.08)	0.17 (0.15)
**LightGBM**	0.54 (0.10)	0.97 (0.03)	0.19 (0.27)
**Dominant hand**			
**Random forest**	0.54 (0.22)	0.96 (0.04)	0.12 (0.06)
**XGBoost**	0.70 (0.24)	1.03 (0.10)	0.12 (0.06)
**LightGBM**	0.60 (0.18)	1.01 (0.04)	0.06 (0.05)

Disparate impact and Equal opportunity are reported as ratios (fair = 1), whereas equalized odds is the difference between unprivileged and privileged groups (fair = 0).

Even after race upsampling, the models exhibited noticeable disparities in predictions associated with device and dominant hand, with DI values ranging from 0.38 to 0.70 and tighter standard deviations than for race or sex (Table 4 and Supplementary Table 11). This indicates that the unprivileged groups (Windows, left hand) are 38–70% less likely to receive a PD diagnosis from the model than the privileged groups (Mac, right hand). Bias metrics assessed before race upsampling, stratified by sex, race, device type, and dominant hand, are detailed in Supplementary Table 12. We observed similar patterns when demographic features were excluded from the model inputs, both before and after race upsampling. Although individual metric values varied slightly, the overall interpretation of the results and patterns related to bias and fairness remained unchanged (Supplementary Tables 13–30).

### Features used in the study

We extracted 79 features from interaction data, encompassing mouse movement trajectories, keyboard press dynamics, and performance metrics from a memory assessment. Supplementary Table 31 summarizes all features used in our analysis to detect PD.

### Stratified feature importance

We conducted both global and stratified analyses (based on device type and handedness) of feature importance to examine which input variables most strongly influenced model predictions. All analyses were performed without race upsampling. At the global level, features derived from mouse movement emerged as the most predictive in distinguishing individuals with PD (Table 5).

**Table 5:** Globally top-ranked features according to a random forest model without race upsampling

Rank	Feature name	Importance	Feature description
1	Total deviation straight line	0.06	Total accumulated deviation from the centerline when tracing a straight line (percentage of screen height)
2	Time to trace a spiral	0.06	Amount of time taken to trace the spiral
3	Net deviation straight line	0.05	Net accumulated deviation from the centerline when tracing a straight line (percentage of screen height)
4	Maximum deviation straight line	0.05	Maximum deviation from the centerline when tracing a straight line (percentage of screen height)
5	Average absolute deviation straight line	0.05	Average of absolute values of deviation from the centerline when tracing a straight line (percentage of screen height)
6	Time to trace a straight line normalized	0.04	Amount of time taken to trace a straight line with respect to the window width
7	Time to trace the spiral normalized	0.04	Amount of time taken to trace the spiral with respect to the window width
8	Maximum pixel deviation straight line	0.03	Maximum deviation from the centerline when tracing a straight line without regard to the window height (pixels)
9	Average tracing time for all tasks	0.03	Average time taken to complete all line-tracing tasks.
10	Time to trace the sine wave normalized	0.03	Amount of time taken to trace the sine wave with respect to the window width

This pattern remained consistent in the subgroup analyses for right-handed participants (Table 6) and those using Windows operating systems (Table 6), where mouse movement features emerged as the most significant indicators of PD. In contrast, PD identification among left-handed participants (Table 6) relied on a combination of mouse movement and keyboard press features. For MacOS users (Table 6), besides the mouse movement data, features across multiple categories, including keyboard press, mouse clicking, and memory-based features, contributed equally to PD detection. Mac and left-handed subgroups often exhibited lower or more variable F1-scores and AUROC values, with Mac users additionally showing substantially reduced specificity. These findings suggest that in the absence of data balancing, models may overfit to idiosyncratic patterns in smaller subgroups, elevating the apparent importance of non-generalizable features. The five most predictive features for each subgroup are presented here, with the complete set of top 10 features provided in Supplementary Table 32.

**Table 6:** Top-ranked features based on dominant hand and device type according to a random forest model without race upsampling

Right-handed			
Rank	Feature name	Importance	Feature description
1	Maximum pixel deviation straight line	0.04	Maximum deviation from the centerline when tracing a straight line without regard to the window height (pixels)
2	Time to trace a straight line	0.04	Amount of time taken to trace a straight line
3	Maximum deviation straight line	0.04	Maximum deviation from the centerline when tracing a straight line (percentage of screen height)
4	Time to trace the sine wave normalized	0.04	Amount of time taken to trace the sine wave with respect to the window width
5	Total deviation straight line	0.04	Total accumulated deviation from the centerline when tracing a straight line (percentage of screen height)
**Left-handed**			
**Rank**	**Feature name**	**Importance**	**Feature description**
1	Correct the press rate constant key	0.08	Ratio of correct key presses to the average response time when prompted with a constant key.
2	Average absolute deviation straight line	0.07	Average of absolute values of deviation from the centerline when tracing a straight line (percentage of screen height)
3	Maximum pixel deviation straight line	0.05	Maximum deviation from the centerline when tracing a straight line without regard to the window height (pixels)
4	Average response time constant key	0.05	Average response time when prompted with a constant key
5	Mean deviation straight line	0.05	Mean deviation from the centerline when tracing a straight line (fraction of screen height)
**Mac**			
**Rank**	**Feature name**	**Importance**	**Feature description**
1	Total click time for all tests	0.06	Total time taken for clicking the box
2	False press ratio random key	0.05	Ratio of false key presses to the total number when prompted with a random key
3	Maximum pixel deviation straight line	0.05	Maximum deviation from the centerline when tracing a straight line without regard to the window height (pixels)
4	Average false presses all tests	0.05	Average false presses from all tests
5	Total click reaction time	0.04	Total reaction time for data collected from the click game
**Windows**			
**Rank**	**Feature name**	**Importance**	**Feature description**
1	Time to trace the sine wave normalized	0.1	Amount of time taken to trace the sine wave with respect to the window width
2	Time to trace a straight line	0.06	Amount of time taken to trace a straight line
3	Average points inside all lines	0.06	Average number of mouse trace points falling within the line boundaries, regardless of the time taken.
4	Time to trace a straight line normalized	0.05	Amount of time taken to trace a straight line with respect to the window width
5	Time to trace a spiral	0.05	Amount of time taken to trace the spiral

After race upsampling, the overall feature importance patterns remained largely consistent with those observed prior to upsampling. Notably, “Device type” newly emerged as a top-ranked global feature and also appeared as an important feature in the left-handed subgroup (Supplementary Tables 33–37).

## Discussion

We build upon a rich literature of existing digital PD assessments that have focused on speech^4^^,18^^,19^^,^ gait^8^, and handwriting^20^ data modalities. Our approach aligns with an increasing interest in self-administered and non-invasive telemonitoring applications, which offer an accessible and convenient alternative to clinical examinations that are often challenging for elderly patients^21,22^. While prior studies have explored keyboard typing and touchscreen interactions for PD classification^23,24^ and multimodal classification^25^ in a similar manner as our approach, few if any have systematically addressed the issue of machine learning bias stemming from small sample sizes for underrepresented groups^26^.

Our analyses uncovered performance disparities concerning device type and dominant hand. These findings emphasize the importance of evaluating potential biases, including less visible ones, in consumer digital health tools aimed at promoting accessible screening.

Our results highlight the need for careful selection of AI bias metrics. Despite showing disparities toward privileged groups in terms of receiving a PD diagnosis for device type and dominant hand, the models exhibited strong performance in terms of EO across all demographic groups, with values ranging between 0.87 and 1.22. However, this metric only captures the TPR (sensitivity) across groups and does not account for false positive rates or overall fairness. The EOdds metric, on the other hand, highlighted notable imbalances across groups.

In some cases, F1-scores were higher for one group than another, but AUROC showed the opposite trend. This discrepancy often occurs when a group’s specificity decreases significantly, affecting AUROC more than the F1-score. Conversely, there were instances where AUROC scores were higher despite lower F1-scores. In these cases, variations in precision mirrored those in F1-scores, while specificity and sensitivity remained strong, contributing to the higher AUROC values.

Stratified analyses by device type and handedness revealed subgroup-specific differences in the features most predictive of PD. For right-handed participants and individuals using Windows devices, which are the majority groups for handedness and device type, the top-ranked features were exclusively derived from line tracing tasks and largely aligned with the global top-ranked features. In contrast, among left-handed participants, four of the ten most important features were associated with key press dynamics. For macOS users, the most influential features spanned multiple modalities, including keyboard press (*n* = 3), mouse clicks (*n* = 2), memory-related performance (*n* = 2), and mouse movement. This broader distribution may either reflect true differences or simply reflect an artifact of overfitting on the smaller subgroups. Because the feature importance analyses were conducted on models trained before race upsampling, the observed subgroup-specific differences must be interpreted with caution. The subsequent improvements in performance observed after race upsampling, particularly in specificity and AUROC for Mac users and overall stability for left-handed participants, further support this interpretation. These results suggest that imbalances along one axis (e.g., race) can exacerbate apparent disparities across others (e.g., device type, handedness) and highlight the importance of using balanced data across many dimensions in consumer digital health.

We note a few prominent limitations of our study^27^, many of which have been addressed in prior literature, that should be addressed in subsequent work pertaining to digital health equity issues in clinical-grade tools (we do not present a clinical-grade tool in this work). First and foremost, we asked participants to self-report their PD diagnoses based on the suggestion from our patient partner that adults are unlikely to lie about a PD diagnosis. Nevertheless, this could have led to noisier data labels. We also collapsed “suspected PD” and “PD” into one diagnostic category for simplicity, which would not be acceptable in a clinical grade PD diagnostic. Another significant limitation is that our model relied on data collected at a single point in time, thus failing to capture the progression of PD over time or symptomatic fluctuations. As PD is a degenerative condition, symptoms can vary due to factors such as medication, stress, and daily activities. There is also a phenotypic progression of PD that we did not account for but that has been modeled in prior work^28^. Additionally, our study does not include detailed symptom quantification or information about participants’ medication usage or timing, critical factors that can significantly influence symptom severity and, thus, model predictions. We did not control for these factors. In future work, we aim to address these limitations by collecting longitudinal data and controlling medication timing and dose. In these future studies, it will be necessary to consider differences in symptom presentation variability across demographic groups, with the temporal aspect potentially introducing additional biases that were not explored in this work.

Another limitation of our approach is that we did not directly measure specific input methods (e.g., mouse vs. trackpad) or dynamic browser performance (e.g., frame rate, latency). These may influence motor input signals and serve as more direct technical covariates than device type alone. However, such data are not consistently available through the web APIs, and given the older age distribution of the study participants, we decided that it would be infeasible to ask participants to self-report this information. Because we could easily and automatically record the device type through our website implementation, we used this attribute as a practical and interpretable proxy for technical heterogeneity. Future work in the field of consumer digital health should incorporate richer instrumentation of user input hardware and system performance metrics to more precisely characterize technical sources of bias.

A further limitation is that, in accordance with epidemiological trends, we primarily recruited individuals aged 50 years and older to align with the population most commonly affected by PD, given that the vast majority of PD cases occur in individuals aged 50 years and older, with prevalence and incidence rising sharply in this age group^29^. However, a small subset was younger than 50 years, reflecting natural variability in the sampled population. While our recruitment strategy enhances the clinical relevance of our findings for the majority of PD cases, this age distribution may limit the generalizability of our results to younger-onset PD populations, who may present with different motor patterns and disease trajectories.

## Methods

### Study design and participant recruitment

We primarily recruited individuals aged 50 years and older, although a small subset was younger than 50. The recruitment pool comprised both adults diagnosed with PD and healthy controls. To facilitate recruitment, we partnered with local organizations dedicated to PD support and advocacy, including through recruitment efforts at the 2023 and 2024 Hawaii Parkinson’s Association Symposiums.

We invited participants to join the study using a web application accessible through a public link. The application provided detailed instructions about the research and its activities. Before commencing any activities, all participants provided informed consent electronically. All study procedures were approved by the University of Hawaii Institutional Review Board (IRB Protocol #2022-00857) and conducted in accordance with applicable ethical standards and guidelines.

**Fig. 5:**
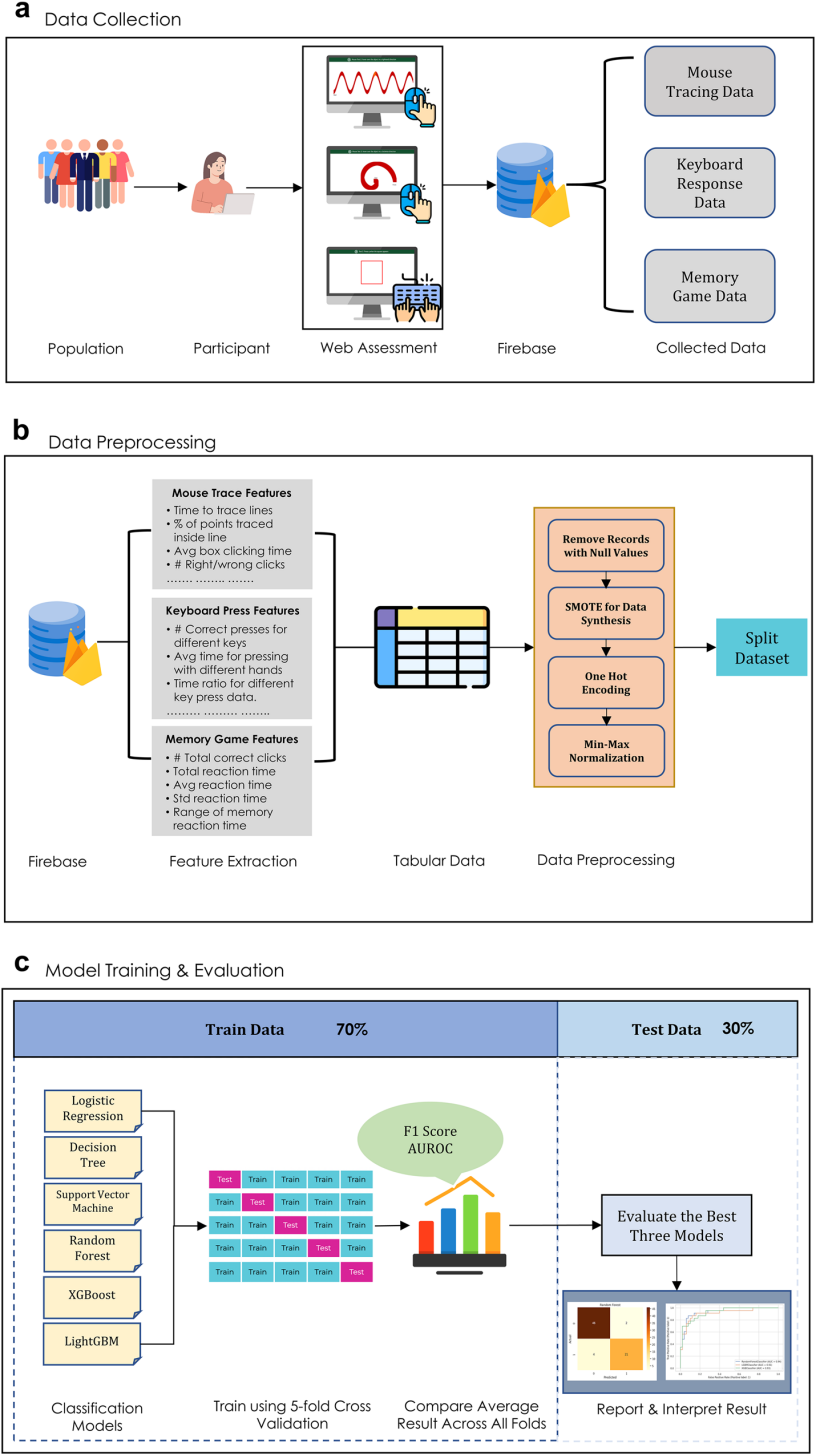
Study workflow. **a** We recorded hand movement data through a structured assessment on a publicly available website. The data are stored in a cloud database for further analysis. **b** Features are extracted from the hand movement data and organized into a tabular format. The features are preprocessed to remove any empty records. The categorical features were transformed using one-hot encoding, and numerical features were normalized using min–max scaling. **c** We trained the model with six machine learning algorithms using 5-fold cross-validation. Icons used in this figure were made by Freepik, Aranagraphics, Pixel Perfect, Vitaliy Gorbachev, Flat Icons Design, HAJICON from www.flaticon.com, used under the Flaticon Free License. The figure also includes icons created with the assistance of OpenAI’s ChatGPT (chat.openai.com) using generative AI. Use of these assets complies with OpenAI’s terms, which permit reuse for publication and commercial purposes.

**Fig. 6:**
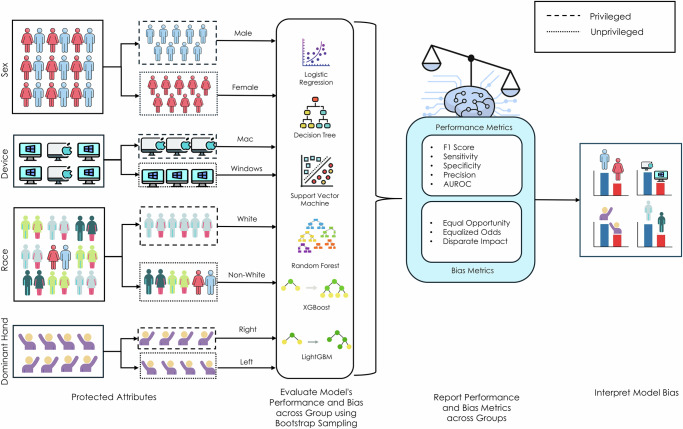
Bias evaluations. We quantified biases concerning sex, race, device type, and handedness. Icons used in this figure were made by Freepik, Aranagraphics, Pixel Perfect, Vitaliy Gorbachev, Flat Icons Design, HAJICON from www.flaticon.com, used under the Flaticon Free License. The figure also includes icons created with the assistance of OpenAI’s ChatGPT (chat.openai.com) using generative AI. Use of these assets complies with OpenAI’s terms, which permit reuse for publication and commercial purposes.

Figure 5 shows the overall procedure for collecting participant data, preprocessing the dataset to train the models, and evaluating the models for real-world applications. The participants used the website to complete a series of assessments. The first assessments, evaluating motor control, required participants to use the mouse to click and trace different objects and press keyboard buttons at various times during the game. The final assessment evaluated working memory by requiring participants to memorize increasingly longer numerical sequences. These data were stored in a cloud database along with the participant’s demographic data (Fig. 5a). The stored data were then analyzed and engineered to extract features from the mouse trace and keyboard press data. Finally, the dataset was randomly split into 70% training and 30% testing data (Fig. 5b). Six common machine learning classification algorithms were used, with 5-fold cross-validation on 70% of the data partitioned for training, and the results were compared for further testing and evaluation on held-out data (Fig. 5c). All models were then tested for performance disparities across race, sex, device type, and handedness (Fig. 6).

### Data collection procedure

**Fig. 7:**
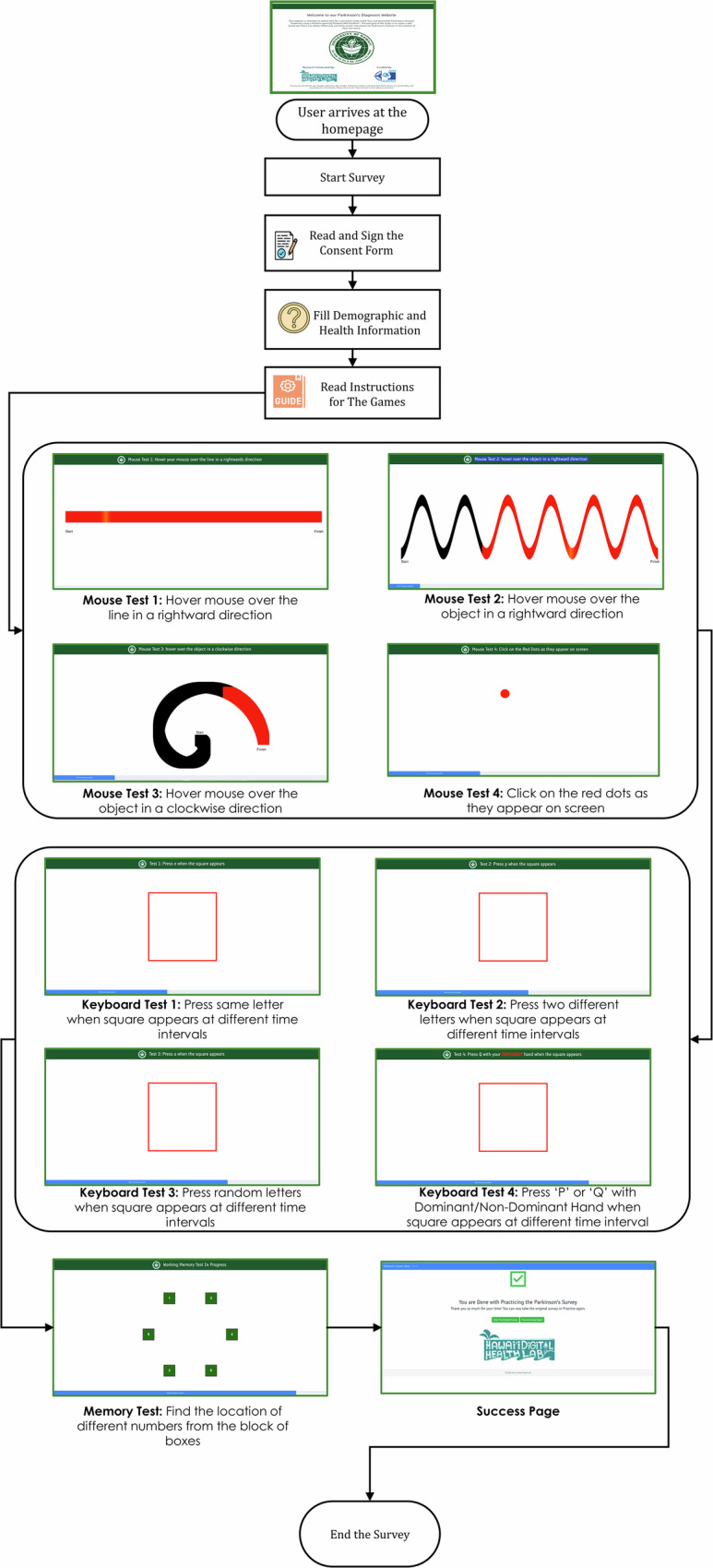
User interface of the web assessment. The user starts by filling out a consent form and records their demographic, health, and medication information. The users are then presented with shapes and required to trace the shape with their mouse/trackpad. This is followed by a series of assessments where the user is asked to press a key on the keyboard immediately when prompted. The game ends with a memory test that we did not incorporate into our analysis. Icons used in this figure were made by Uniconlabs, Ehtisham Abid and Peerapak Takpho from www.flaticon.com, used under the Flaticon Free License.

We recorded the participant data using a web-based assessment by evaluating both motor functions and a working memory test (Fig. 7). This evaluation was conducted through short and structured assessments provided sequentially. We used established assessment paradigms to enable scalable, noninvasive digital phenotyping aligned with established PD symptom domains.

The mouse and keyboard assessments were designed to capture digital correlates of motor and cognitive impairments commonly observed in PD. Spiral and line tracing tasks are widely used to assess bradykinesia and fine motor control and have been validated as digital biomarkers of PD severity^30,31^. Similarly, timed keypress and keyboard sequence tasks have been shown to reflect bradykinetic motor patterns and executive function deficits^31,32^.

The mouse-based assessments included:

1. Straight-line tracing: Participants were tasked with tracing straight lines on the screen to evaluate hand stability and precision. This task required participants to maintain consistent control and accuracy, reflecting fine motor skills.
2. Curved line tracing: Participants traced spirals and other curved shapes during this activity, offering a more complex evaluation of hand stability and motor coordination.
3. Randomized clicking tasks: Participants clicked on targets appearing at random locations on the screen to measure reaction time, hand-eye coordination, and accuracy.

The keyboard-based assessments consisted of:

1. Single-letter presses: participants were instructed to press designated keys immediately upon receiving a visual prompt, assessing their motor response speed and accuracy.
2. Multiple-letter presses: this task involved replicating key sequences, evaluating participants’ ability to execute complex motor tasks and process sequential information. In this phase, the task was confined to only two letters, and the user only needed to press one of the two letters that appeared on the screen to pass.
3. Randomized Sequences: participants completed tasks involving sequences of randomized key presses, testing their adaptability, coordination, and cognitive flexibility.

We also added a memory assessment to measure cognitive function through progressively challenging levels. Players were tasked with memorizing a sequence of numbers and then recapitulating the sequence. This task is conceptually similar to the digit span test, which is commonly used in Parkinson’s Disease research to evaluate working memory and executive function as part of broader cognitive screening batteries^33^.

### Data up-sampling

**Fig. 8:**
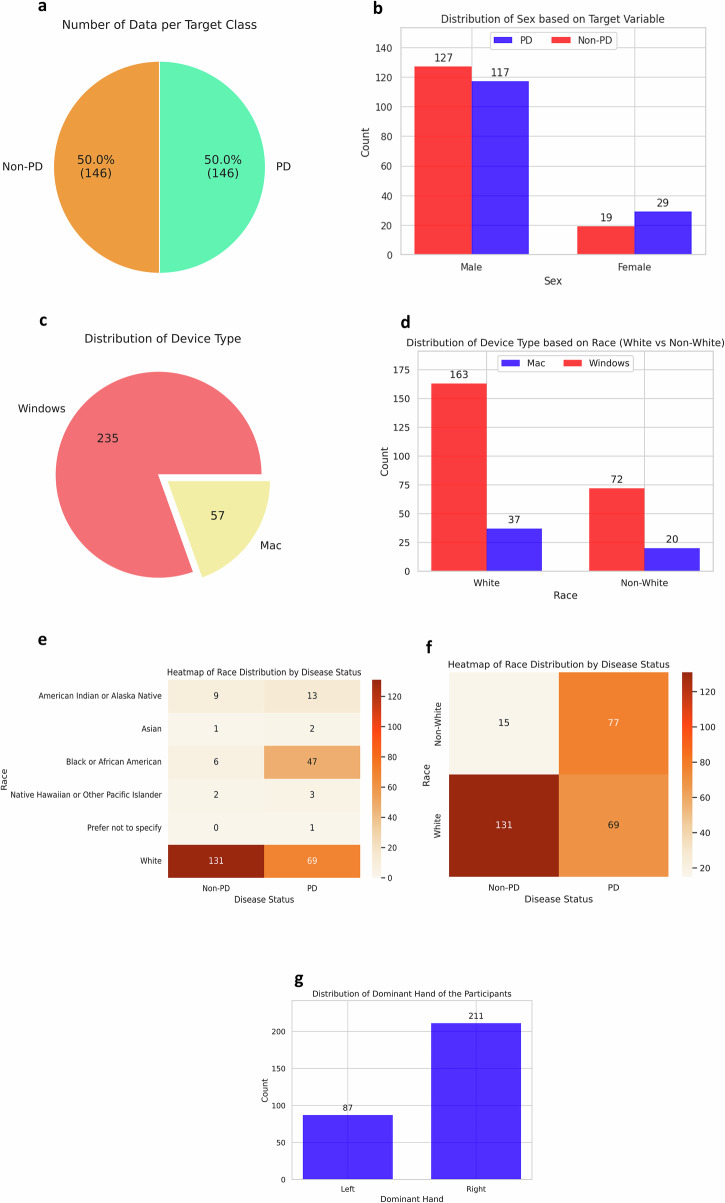
Data distribution after applying SMOTE. Demographic and technical distribution of the dataset used for Parkinson’s Disease (PD) classification. **a** Class balance of PD and Non-PD participants. **b** Sex distribution stratified by PD status. **c** Device type distribution. **d** Device usage by racial group. **e**, **f** Heatmaps of race distribution across PD status. **g** Distribution of dominant hand among participants.

The initial dataset used in our study comprised 152 healthy individuals (60.6%) and 99 participants who self-reported a diagnosis of PD or suspected PD (39.4%). While the model initially achieved a strong F1-score, its sensitivity remained low, around 50%, which is particularly concerning in healthcare applications where detecting true positive cases is crucial. To address this issue and to improve the sensitivity of the models, we applied the synthetic minority over-sampling technique (SMOTE) to balance the dataset by up-sampling the PD patient data (updated data distribution shown in Fig. 8). Given that only eight participants used Linux, this group was excluded during up-sampling to maintain a balanced representation across device types.

**Fig. 9:**
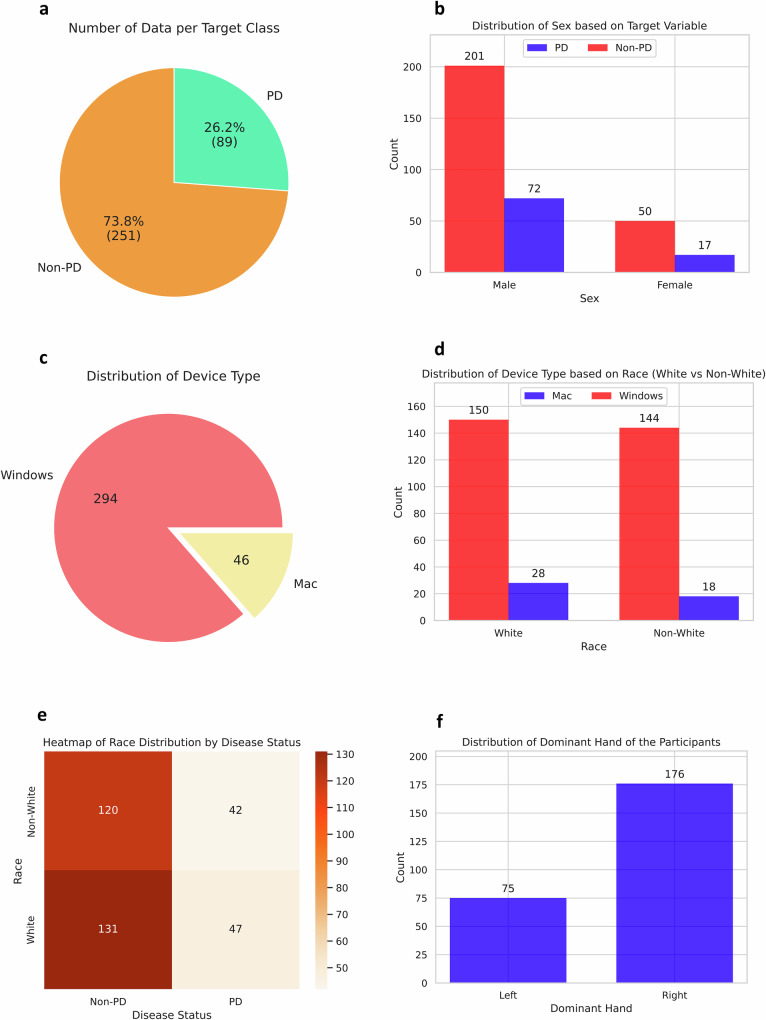
Data distribution after randomly resampling Non-White data points with no PD (before SMOTE). **a** Class balance of PD and Non-PD participants. **b** Sex distribution stratified by PD status. **c** Device type distribution. **d** Device usage by racial group. **e** Heatmaps of race distribution across PD status. **f** Distribution of dominant hand among participants.

**Fig. 10:**
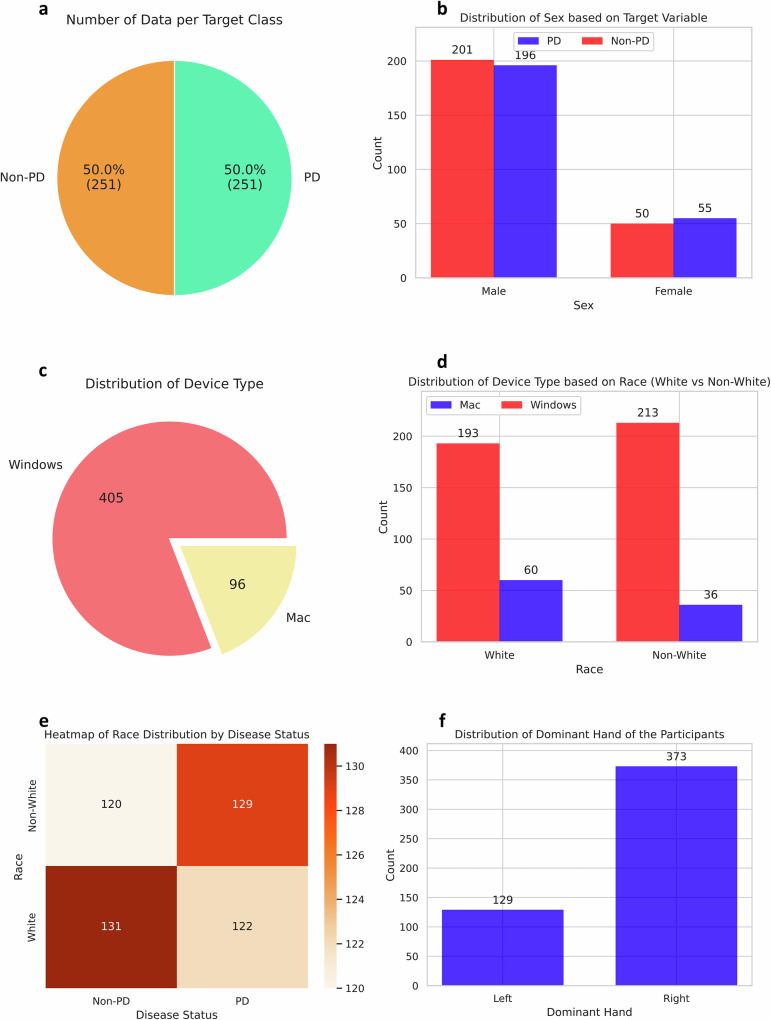
Data distribution after randomly resampling non-White data points with no PD (after SMOTE). **a** Class balance of PD and Non-PD participants. **b** Sex distribution stratified by PD status. **c** Device type distribution. **d** Device usage by racial group. **e** Heatmaps of race distribution across PD status. **f** Distribution of dominant hand among participants.

Moreover, there was a significant imbalance in the racial distribution within the PD label, with 131 White participants compared to only 15 Non-White participants. To mitigate this imbalance, we applied a random resampling technique to increase the number of Non-White participants in the PD category (updated data distribution without SMOTE shown in Fig. 9 and with SMOTE shown in Fig. 10).

### Bootstrapped sampling

We apply bootstrapped sampling with 100 samples in each iteration to generate error bars. In each bootstrapped sampling step, the sample set consists of an equal number of data points from both groups in each category while calculating the group-wise metrics for that category, e.g., sex, race, device, and dominant hand. For example, when calculating the F1-scores, sensitivity, specificity, precision, and AUROC for sex, the sample set was constructed by taking 50% of the sample from the Male subset and 50% from the Female subset using resampling with replacement. Finally, we report the mean of the metrics.

### Post-hoc power analysis

To justify our sample size, we provide a post-hoc power analysis using a standard power calculator for binary classification of disease: https://sample-size.net/sample-size-ci-for-auroc/. We input AUROC = 0.91, which is the lowest AUROC we get on a held-out test set out of our top-3 performing models (higher AUROCs lead to lower sample size requirements, so this was a conservative choice). We input 0.39 for the proportion having the disease, which aligns with the dataset we collected for this study. We use the standard 0.125 for the confidence interval width and 0.95 for the confidence level. Using these parameters, we find that our study requires a minimum sample size of 111 to be statistically justifiable. The sample size used in our study is over twice this size (*n* = 251), thus indicating that our study is adequately powered.

### Evaluation metrics

To evaluate the performance of our PD prediction model, we used a combination of standard performance metrics and fairness metrics to assess both accuracy and equity across demographic groups. This section provides a detailed description of these metrics.

We compute the F1-score, accuracy, sensitivity (recall), precision, and specificity to evaluate model performance (Eqs. 1, 2, 3, 4, and 5). Precision represents the proportion of correctly identified positive cases out of all predicted positive cases. Sensitivity (recall) measures the proportion of true positive (TP) cases accurately determined by the model. At the same time, specificity quantifies the proportion of true negatives (TN) cases correctly classified, where true negatives refer to actual negatives correctly identified. In these calculations, false positives (FP) represent cases incorrectly classified as positive, and false negatives (FN) are actual positives misclassified as unfavorable. The F1-score combines precision and recall into a single metric, calculated as their weighted harmonic mean. Accuracy captures the overall correctness of the model’s predictions.

The formulas for these metrics are:

1 $${Precision}=\frac{{TP}}{{TP}+{FP}}$$

2 $${Recall}=\frac{{TP}}{{TP}+{FN}}$$

3 $${Specificity}=\frac{{TN}}{{TN}+{FP}}$$

4 $$F1-{score}=\frac{2* ({Recall}* {Precision})}{({Recall}+{Precision})}$$

5 $${Accuracy}=\frac{{TP}+{TN}}{{TP}+{FP}+{TN}+{FN}}$$

Alongside these metrics, we also report the area under the receiver operating characteristic curve (AUROC), which serves as a threshold-independent measure of the model’s ability to distinguish between positive and negative cases.

To ensure equitable outcomes across protected groups, we employed the following fairness metrics: DI, EO, and EOdds.

DI is a fairness metric used to evaluate equity in model outcomes by comparing the rates of positive outcomes between a privileged and an unprivileged group^34,35^. It assesses whether the unprivileged group is disproportionately less likely to receive favorable outcomes than the privileged group. DI is calculated as the ratio of the proportion of positive outcomes for the unprivileged group to that of the privileged group, defined as Eq. 6.

6 $${DI}=\frac{\left(\frac{T{P}_{u}+F{P}_{u}}{{N}_{u}}\right)}{\left(\frac{T{P}_{P}+F{P}_{P}}{{N}_{P}}\right)}$$

${{TP}}_{u}$ and ${{TP}}_{p}$ represent true positives, while ${{FP}}_{u}$ and ${{FP}}_{p}$ denote false positives for the unprivileged (*u*) and privileged (*p*) groups. ${N}_{u}$ and ${{N}}_{p}$ denote the total number of individuals in the unprivileged and privileged groups, respectively.

To achieve fairness, the DI value must equal 1, indicating equal rates of positive predictions for both groups^32,33^. A value below 1 signals potential bias against the unprivileged group, while a value above 1 suggests a potential bias favoring the unprivileged group.

EO is a fairness concept requiring that privileged and unprivileged groups achieve equal true positive rates (TPR)^35,36^. This ensures that individuals from all demographic groups who qualify for a positive outcome are equally likely to receive it. EO is mathematically defined as the ratio of the TPRs between unprivileged and privileged groups and is expressed in Eq.7:

7 $${EO}=\frac{{{TPR}}_{u}}{{{TPR}}_{p}}$$

An EO value of 1 represents perfect fairness, signifying that TPRs are identical across both privileged and unprivileged groups. A value less than 1 indicates that the unprivileged group has a lower TPR than the privileged group, suggesting potential bias against the unprivileged group in receiving favorable outcomes. Conversely, a value greater than 1 indicates that the unprivileged group has a higher TPR than the privileged group, which may reflect a bias favoring the unprivileged group.

EOdds requires that both TPRs and FPRs are equal across privileged and unprivileged groups^36^, ensuring that the model’s predictions regarding successes and errors are fair. By extending the concept of EO, EOdds ensures that predictions do not disproportionately favor or disadvantage any group. It can be mathematically represented as the differences of TPRs and FPRs between the unprivileged and privileged groups showed in Eq. (8)

8 $${EOdds}=max\left({{TPR}}_{u}-\,{{TPR}}_{p},\,{\,{FPR}}_{u}-{{FPR}}_{p}\right)$$

An EOdds value of 0 represents ideal fairness, signifying a uniform error distribution and success rates across all demographic groups. Deviations from this standard indicate disparities in the model’s predictions, pointing to potential biases in TPRs, FPRs, or both.

## Supplementary information

Supplementary information

## Data Availability

We plan to release this dataset as a publicly available dataset through a separate dataset-oriented publication. In the meantime, we have provided a subset of the dataset in the GitHub repository below. Prior to publication of our supplemental dataset paper, readers may contact the first author for dataset access and cite this publication when using the data.

## Code availability

The machine learning code used to perform the analyses in this study is available at https://github.com/ucsfdigitalhealth/quantifying-bias-remote-pd-website. The code is provided with a subset from the original dataset due to our plan to release the dataset in a separate publication, but it includes documentation and synthetic examples to support reproducibility.

## References

[1] Dorsey, E. R., Sherer, T., Okun, M. S. & Bloem, B. R. The emerging evidence of the Parkinson pandemic. *J. Parkinsons Dis.* **8**, S3–S8 (2018). 30584159 10.3233/JPD-181474 PMC6311367

[2] Dorsey, E. R. et al. Projected number of people with Parkinson disease in the most populous nations, 2005 through 2030. *Neurology* **68**, 384–386 (2007). 17082464 10.1212/01.wnl.0000247740.47667.03

[3] Almalaq, A., Dai, X., Zhang, J., Hanrahan, S. & Nedrud, J. Causality graph learning on cortical information flow in Parkinson’s disease patients during behaviour tests. In *Proc. 49th Asilomar Conference on Signals Systems, and Comput**ers* 925–929 (IEEE, 2015).

[4] Benba, A., Jilbab, A., Hammouch, A. & Sandabad, S. Voiceprints analysis using MFCC and SVM for detecting patients with Parkinson’s disease. In *Proc. IEEE International Conference on Electronics and Information Technology (ICEIT)* 300–304 (IEEE, 2015).

[5] Sakar, C. O. et al. A comparative analysis of speech signal processing algorithms for Parkinson’s disease classification and the use of the tunable-factor wavelet transform. *Appl. Soft Comput.* **74**, 255–263 (2019).

[6] Alqahtani, E. J., Alshamrani, F. H., Syed, H. F. & Olatunji, S. O. Classification of Parkinson’s disease using NNge classification algorithm. In *Proc. 21st Saudi Computer Society National Computer Conference (NCC)* 1–7 (IEEE, 2018).

[7] Pereira, C. R. et al. A new computer vision-based approach to aid the diagnosis of Parkinson’s disease. *Comput. Biol. Med.* **136**, 79–88 (2016). 10.1016/j.cmpb.2016.08.005 27686705

[8] Abdulhay, E. et al. Gait and tremor investigation using machine learning techniques for the diagnosis of Parkinson’s disease. *Future Gener. Comput. Syst.* **83**, 366–373 (2018).

[9] Asanza, V. et al. Classification of subjects with Parkinson’s disease using finger tapping dataset. *IFAC-PapersOnLine* **54**, 376–381 (2021).

[10] Adams, W. R. High-accuracy detection of early Parkinson’s disease using multiple characteristics of finger movement while typing. *PLoS One* **12**, e0188226 (2017). 29190695 10.1371/journal.pone.0188226 PMC5708704

[11] Parab, S., Boster, J. & Washington, P. Parkinson's disease recognition using a gamified website: machine learning development and usability study. *JMIR Form. Res.* **7**, e49898 (2023). 37773607 10.2196/49898 PMC10576230

[12] Kassavetis, P. et al. Developing a tool for remote digital assessment of Parkinson’s disease. *Mov. Disord. Clin. Pract.* **3**, 59–64 (2016). 30363542 10.1002/mdc3.12239 PMC6178716

[13] Papapetropoulos, S., Mitsi, G. & Espay, A. J. Digital health revolution: is it time for affordable remote monitoring for Parkinson’s disease?. *Front. Neurol.* **6**, 34 (2015). 25767462 10.3389/fneur.2015.00034 PMC4341545

[14] Gatsios, D. et al. Feasibility and utility of mHealth for the remote monitoring of Parkinson’s disease: ancillary study of the PD_manager randomized controlled trial. *JMIR Mhealth Uhealth* **8**, e16414 (2020). 32442154 10.2196/16414 PMC7367523

[15] Negi, A. S. et al. Remote real-time digital monitoring fills a critical gap in the management of Parkinson’s disease. *medRxiv*. 10.1101/2024.12.12.24318893 (2024). 10.1038/s41531-025-01101-0 PMC12344058 40796568

[16] Hoffman, S. L. et al. Comprehensive real-time remote monitoring for Parkinson’s disease using quantitative DigitoGraphy. *npj Parkinsons Dis.* **10**, 137 (2024). 39068150 10.1038/s41531-024-00751-w PMC11283542

[17] Zawad, M. R. S., Tumpa, Z. N., Sollis, L., Parab, S. & Washington, P. Deep learning prediction of Parkinson’s disease using remotely collected structured mouse trace data. *medRxiv*10.1101/2024.10.27.24316195 (2024).

[18] Neharika, D. B. & Anusuya, S. Machine learning algorithms for detection of Parkinson’s disease using motor symptoms: speech and tremor. *Int. J. Recent Technol. Eng.* **8**, 47–50 (2020).

[19] Sakar, B. E. et al. Collection and analysis of a Parkinson speech dataset with multiple types of sound recordings. *IEEE J. Biomed. Health Inform.* **17**, 828–834 (2013). 25055311 10.1109/JBHI.2013.2245674

[20] Al-Wahishi, A., Belal, N. & Ghanem, N. Diagnosis of Parkinson’s disease by deep learning techniques using handwriting dataset. In *Proc. International Symposium on Signal Processing and Intelligent Recognition Systems (SIRS)*. 10.1007/978-981-16-0425-6_10 (Springer, 2020).

[21] Isenkul, M., Sakar, B. & Kursun, O. Improved spiral test using digitized graphics tablet for monitoring Parkinson’s disease. In *Proc. 2nd International Conference on e-Health Telemedicine (ICEHTM)* **5** (ACM, 2014).

[22] Matarazzo, M. et al. Remote monitoring of treatment response in Parkinson’s disease: the habit of typing on a computer. *Mov. Disord.* **34**, 1488–1495 10.1002/mds.27772 (2019). 31211469 10.1002/mds.27772

[23] Arroyo-Gallego, T. et al. Detecting motor impairment in early Parkinson’s disease via natural typing interaction with keyboards: validation of the neuroQWERTY approach in an uncontrolled at-home setting. *J. Med. Internet Res.* **20**, e89 10.2196/jmir.9462 (2018). 29581092 10.2196/jmir.9462 PMC5891671

[24] Bernardo, L. S. et al. Modified SqueezeNet architecture for Parkinson’s disease detection based on keypress data. *Biomedicines* **10**, 2746 (2022). 36359266 10.3390/biomedicines10112746 PMC9687688

[25] Makarious, M. B. et al. Multi-modality machine learning predicting Parkinson’s disease. *npj Parkinsons Dis.* **8**, 35 (2022). 35365675 10.1038/s41531-022-00288-w PMC8975993

[26] Paul, S. et al. Bias investigation in artificial intelligence systems for early detection of Parkinson’s disease: a narrative review. *Diagnostics* **12**, 166 (2022). 35054333 10.3390/diagnostics12010166 PMC8774851

[27] Sun, Y. et al. Digital biomarkers for precision diagnosis and monitoring in Parkinson’s disease. *npj Digit. Med.* **7**, 218 (2024). 39169258 10.1038/s41746-024-01217-2 PMC11339454

[28] Su, C. et al. Identification of Parkinson’s disease PACE subtypes and repurposing treatments through integrative analyses of multimodal data. *npj Digit. Med.* **7**, 184 (2024). 38982243 10.1038/s41746-024-01175-9 PMC11233682

[29] Willis, A. W. et al. Incidence of Parkinson disease in North America. *npj Parkinsons Dis.* **8**, 170 (2022). 36522332 10.1038/s41531-022-00410-y PMC9755252

[30] Heldman, D. ustinA. et al. The modified Bradykinesia rating scale for Parkinson’s disease: reliability and comparison with kinematic measures. *Mov. Disord.* **26**, 1859–1863 (2011). 21538531 10.1002/mds.23740 PMC3324112

[31] Hasan et al. Technologies assessing limb Bradykinesia in Parkinson’s disease. *J. Parkinson’s. Dis.* **7**, 65–7y (2017). 28222539 10.3233/JPD-160878 PMC5302048

[32] Pal, G. ian & Goetz, C. hristopherG. Assessing Bradykinesia in Parkinsonian disorders. *Front. Neurol.* **4**, 54 (2013). 23760683 10.3389/fneur.2013.00054 PMC3669893

[33] Litvan, I. rene et al. Diagnostic criteria for mild cognitive impairment in Parkinson’s disease: movement disorder society task force guidelines. *Mov. Disord.* **27**, 349–356 (2012). 22275317 10.1002/mds.24893 PMC3641655

[34] Briscoe, J. & Gebremedhin, A. Facets of disparate impact: evaluating legally consistent bias in machine learning. In *Proc. 33rd ACM International Conference on Information and Knowledge Management* (ACM Digital Library, 2024).

[35] Caton, S. & Haas, C. Fairness in machine learning: a survey. *ACM Comput. Surv.* **56**, 1–38 (2024).

[36] Mehrabi, N. et al. A survey on bias and fairness in machine learning. *ACM Comput. Surv.* **54**, 1–35 (2021).

