# Supplementary material for: Quantifying device type and handedness biases in a remote Parkinson’s disease AI-powered assessment
Source: NPJ Digit Med. 2025 Aug 27;8:550. doi: 10.1038/s41746-025-01934-2 (PMC12391457; doi:10.1038/s41746-025-01934-2)
Supplement: Supplementary file 1 — Supplementary information [file 41746_2025_1934_MOESM1_ESM.pdf]

Supplementary Information

Supplementary Table 1: Performance of the remaining models after race upsampling on each group-based sex, race, device type and dominant type

	F1-score		Sensitivity		Specificity		Precision		AUROC	
**Sex**	**Male**	**Female**	**Male**	**Female**	**Male**	**Female**	**Male**	**Female**	**Male**	**Female**
Logistic Regression	0.87 (0.03)	0.86 (0.12)	0.92 (0.07)	0.91 (0.13)	0.85 (0.04)	0.77 (0.25)	0.84 (0.02)	0.84 (0.15)	0.93 (0.01)	0.85 (0.16)
Decision Tree	0.91 (0.05)	0.82 (0.12)	0.92 (0.08)	0.93 (0.13)	0.92 (0.03)	0.66 (0.28)	0.91 (0.03)	0.76 (0.16)	0.95 (0.04)	0.78 (0.23)
Support Vector Machine	0.88 (0.03)	0.88 (0.08)	0.89 (0.06)	0.90 (0.09)	0.88 (0.04)	0.82 (0.20)	0.87 (0.04)	0.89 (0.11)	0.93 (0.02)	0.87 (0.13)
**Race**	**White**	**Non-White**	**White**	**Non-White**	**White**	**Non-White**	**White**	**Non-White**	**White**	**Non-White**
Logistic Regression	0.80 (0.10)	0.90 (0.08)	0.83 (0.08)	0.95 (0.04)	0.48 (0.40)	0.55 (0.45)	0.81 (0.16)	0.88 (0.15)	0.88 (0.02)	0.97 (0.02)
Decision Tree	0.88 (0.11)	0.89 (0.07)	0.92 (0.08)	0.90 (0.10)	0.51 (0.42)	0.56 (0.46)	0.84 (0.14)	0.90 (0.12)	0.89 (0.04)	0.98 (0.03)
Support Vector Machine	0.80 (0.06)	0.89 (0.08)	0.78 (0.05)	0.93 (0.03)	0.51 (0.42)	0.55 (0.45)	0.84 (0.13)	0.87 (0.15)	0.89 (0.02)	0.97 (0.02)
**Device**	**Mac**	**Windows**	**Mac**	**Windows**	**Mac**	**Windows**	**Mac**	**Windows**	**Mac**	**Windows**
Logistic Regression	0.87 (0.09)	0.85 (0.07)	0.98 (0.04)	0.88 (0.10)	0.21 (0.25)	0.89 (0.05)	0.79 (0.15)	0.84 (0.07)	0.66 (0.30)	0.95 (0.04)
Decision Tree	0.94 (0.04)	0.86 (0.08)	0.92 (0.07)	0.90 (0.10)	0.80 (0.25)	0.89 (0.04)	0.97 (0.04)	0.83 (0.06)	0.91 (0.10)	0.93 (0.06)
Support Vector Machine	0.86 (0.09)	0.85 (0.05)	0.97 (0.06)	0.84 (0.06)	0.19 (0.23)	0.92 (0.03)	0.79 (0.15)	0.87 (0.04)	0.54 (0.26)	0.95 (0.03)
**Dominant Hand**	**Right**	**Left**	**Right**	**Left**	**Right**	**Left**	**Right**	**Left**	**Right**	**Left**
Logistic Regression	0.89 (0.03)	0.79 (0.13)	0.93 (0.08)	0.88 (0.11)	0.85 (0.02)	0.90 (0.08)	0.86 (0.02)	0.76 (0.21)	0.93 (0.02)	0.96 (0.03)
Decision Tree	0.89 (0.05)	0.88 (0.10)	0.91 (0.08)	0.95 (0.06)	0.87 (0.04)	0.94 (0.06)	0.88 (0.04)	0.84 (0.14)	0.93 (0.05)	0.93 (0.08)
Support Vector Machine	0.89 (0.02)	0.79 (0.14)	0.90 (0.05)	0.88 (0.11)	0.88 (0.03)	0.91 (0.07)	0.88 (0.04)	0.78 (0.22)	0.93 (0.02)	0.96 (0.04)

Supplementary Table 2: Model performance before race upsampling on each group based on sex, race, device type and dominant hand

	F1-score		Sensitivity		Specificity		Precision		AUROC	
**Sex**	**Male**	**Female**	**Male**	**Female**	**Male**	**Female**	**Male**	**Female**	**Male**	**Female**
Logistic Regression	0.87 (0.03)	0.80 (0.18)	0.88 (0.10)	0.90 (0.13)	0.89 (0.06)	0.67 (0.31)	0.87 (0.07)	0.80 (0.25)	0.93 (0.04)	0.76 (0.19)
Decision Tree	0.82 (0.07)	0.67 (0.22)	0.84 (0.12)	0.76 (0.27)	0.84 (0.06)	0.55 (0.28)	0.82 (0.05)	0.69 (0.24)	0.85 (0.07)	0.74 (0.12)
Support Vector Machine	0.87 (0.03)	0.80 (0.18)	0.89 (0.09)	0.85 (0.20)	0.89 (0.05)	0.81 (0.25)	0.87 (0.05)	0.84 (0.24)	0.93 (0.04)	0.78 (0.24)
Random Forest	0.93 (0.03)	0.83 (0.15)	0.95 (0.05)	0.97 (0.06)	0.92 (0.05)	0.62 (0.21)	0.91 (0.06)	0.76 (0.21)	0.97 (0.03)	0.94 (0.08)
XGBoost	0.82 (0.12)	0.79 (0.13)	0.93 (0.07)	0.97 (0.06)	0.69 (0.35)	0.58 (0.13)	0.77 (0.20)	0.69 (0.18)	0.96 (0.03)	0.90 (0.09)
LightGBM	0.88 (0.04)	0.85 (0.11)	0.91 (0.08)	0.93 (0.08)	0.88 (0.08)	0.71 (0.17)	0.87 (0.08)	0.80 (0.16)	0.96 (0.05)	0.92 (0.08)
**Race**	**White**	**Non-White**	**White**	**Non-White**	**White**	**Non-White**	**White**	**Non-White**	**White**	**Non-White**
Logistic Regression	0.71 (0.14)	0.92 (0.06)	0.74 (0.12)	0.97 (0.03)	0.92 (0.07)	0.27 (0.39)	0.77 (0.23)	0.89 (0.11)	0.92 (0.02)	0.88 (0.09)
Decision Tree	0.68 (0.10)	0.86 (0.06)	0.80 (0.15)	0.82 (0.07)	0.82 (0.05)	0.56 (0.33)	0.64 (0.14)	0.91 (0.09)	0.83 (0.08)	0.70 (0.06)
Support Vector Machine	0.73 (0.09)	0.93 (0.05)	0.75 (0.12)	0.97 (0.03)	0.92 (0.04)	0.29 (0.40)	0.77 (0.14)	0.90 (0.09)	0.91 (0.01)	0.87 (0.12)
Random Forest	0.84 (0.11)	0.94 (0.04)	0.93 (0.04)	0.98 (0.03)	0.92 (0.06)	0.44 (0.39)	0.80 (0.18)	0.91 (0.09)	0.97 (0.02)	0.86 (0.11)
XGBoost	0.71 (0.23)	0.89 (0.09)	0.94 (0.07)	0.90 (0.06)	0.71 (0.31)	0.46 (0.44)	0.65 (0.30)	0.90 (0.13)	0.95 (0.02)	0.91 (0.05)
LightGBM	0.79 (0.11)	0.91 (0.06)	0.88 (0.07)	0.95 (0.05)	0.91 (0.07)	0.41 (0.40)	0.78 (0.20)	0.90 (0.11)	0.94 (0.04)	0.94 (0.04)
**Device**	**Mac**	**Windows**	**Mac**	**Windows**	**Mac**	**Windows**	**Mac**	**Windows**	**Mac**	**Windows**
Logistic Regression	0.95 (0.05)	0.80 (0.05)	1.00 (0.00)	0.81 (0.13)	0.16 (0.20)	0.89 (0.08)	0.91 (0.09)	0.84 (0.10)	0.72 (0.20)	0.90 (0.03)
Decision Tree	0.83 (0.19)	0.75 (0.04)	0.79 (0.26)	0.81 (0.12)	0.43 (0.39)	0.80 (0.07)	0.96 (0.05)	0.72 (0.05)	0.78 (0.05)	0.85 (0.03)
Support Vector Machine	0.94 (0.04)	0.81 (0.06)	0.98 (0.04)	0.80 (0.13)	0.15 (0.20)	0.91 (0.04)	0.91 (0.08)	0.85 (0.05)	0.51 (0.00)	0.90 (0.04)
Random Forest	0.98 (0.03)	0.87 (0.04)	1.00 (0.00)	0.91 (0.06)	0.43 (0.39)	0.89 (0.08)	0.96 (0.05)	0.86 (0.08)	0.83 (0.09)	0.97 (0.04)
XGBoost	0.96 (0.05)	0.77 (0.12)	0.99 (0.03)	0.92 (0.08)	0.33 (0.42)	0.69 (0.30)	0.94 (0.08)	0.71 (0.20)	0.97 (0.03)	0.94 (0.02)
LightGBM	0.96 (0.03)	0.83 (0.03)	0.96 (0.05)	0.87 (0.08)	0.44 (0.39)	0.87 (0.07)	0.96 (0.05)	0.82 (0.08)	0.92 (0.11)	0.94 (0.04)
**Dominant Hand**	**Right**	**Left**	**Right**	**Left**	**Right**	**Left**	**Right**	**Left**	**Right**	**Left**
Logistic Regression	0.85 (0.02)	0.88 (0.06)	0.88 (0.10)	0.88 (0.09)	0.80 (0.12)	0.98 (0.03)	0.85 (0.08)	0.92 (0.13)	0.90 (0.03)	0.92 (0.04)

Decision Tree	0.80 (0.05)	0.64 (0.18)	0.81 (0.10)	0.73 (0.20)	0.78 (0.08)	0.85 (0.12)	0.82 (0.05)	0.66 (0.24)	0.82 (0.03)	0.77 (0.09)
Support Vector Machine	0.86 (0.03)	0.86 (0.07)	0.88 (0.10)	0.86 (0.10)	0.84 (0.08)	0.97 (0.04)	0.87 (0.07)	0.88 (0.12)	0.90 (0.03)	0.93 (0.03)
Random Forest	0.91 (0.02)	0.86 (0.14)	0.94 (0.03)	0.92 (0.08)	0.85 (0.09)	0.95 (0.07)	0.89 (0.05)	0.84 (0.20)	0.97 (0.03)	0.97 (0.03)
XGBoost	0.84 (0.07)	0.67 (0.32)	0.92 (0.06)	0.92 (0.10)	0.67 (0.27)	0.72 (0.35)	0.80 (0.13)	0.64 (0.38)	0.93 (0.04)	0.96 (0.03)
LightGBM	0.88 (0.03)	0.74 (0.17)	0.90 (0.03)	0.88 (0.10)	0.84 (0.09)	0.90 (0.10)	0.88 (0.06)	0.70 (0.25)	0.93 (0.04)	0.95 (0.04)

Supplementary Table 3: Binary indicator of whether a statistically significant difference in F1-score is observed using a t-test between groups for each sensitive attribute (after race upsampling).

		**Logistic Regression**	**Decision Tree**	**Support Vector Machine**	**Random Forest**	**XGBoost**	**LightGBM**
**Statistically Significant F1-score Differences**	**Sex**	No	No	No	No	No	No
	**Race**	No	No	No	No	No	No
	**Device**	No	No	No	Yes	No	No
	**Dominant Hand**	No	No	No	No	No	No

Supplementary Table 4: Binary indicator of whether a statistically significance difference in precision is observed using a t-test between groups for each sensitive attribute (after race upsampling).

		**Logistic Regression**	**Decision Tree**	**Support Vector Machine**	**Random Forest**	**XGBoost**	**LightGBM**
**Statistically Significant Precision Score Differences**	**Sex**	No	No	No	No	No	No
	**Race**	No	No	No	No	No	No
	**Device**	No	Yes	No	Yes	No	No
	**Dominant Hand**	No	No	No	No	No	No

Supplementary Table 5: Binary indicator of whether a statistically significance difference in sensitivity is observed using a t-test between groups for each sensitive attribute (after race upsampling).

		**Logistic Regression**	**Decision Tree**	**Support Vector Machine**	**Random Forest**	**XGBoost**	**LightGBM**
**Statistically Significant Sensitivity**	**Sex**	No	No	No	No	No	No
	**Race**	Yes	No	Yes	No	No	No
	**Device**	No	No	Yes	No	No	Yes

Score Differences	Dominant Hand	No	No	No	No	No	No

Supplementary Table 6: Binary indicator of whether a statistically significance difference in specificity is observed using a t-test between groups for each sensitive attribute (after race upsampling).

		Logistic Regression	Decision Tree	Support Vector Machine	Random Forest	XGBoost	LightGBM
Statistically Significant Specificity Score Differences	Sex	No	No	No	No	No	No
	Race	Yes	No	Yes	No	No	No
	Device	No	No	Yes	No	No	Yes
	Dominant Hand	No	No	No	No	No	No

Supplementary Table 7: Binary indicator of whether a statistical difference in F1-score is observed using a t-test between groups for each sensitive attribute (before race upsampling).

		Logistic Regression	Decision Tree	Support Vector Machine	Random Forest	XGBoost	LightGBM
Statistically Significant F1-score Differences	Sex	No	No	No	No	No	No
	Race	Yes	Yes	Yes	No	No	No
	Device	Yes	No	Yes	Yes	Yes	Yes
	Dominant Hand	No	No	No	No	No	No

Supplementary Table 8: Binary indicator of whether a statistically significance difference in precision is observed using a t-test between groups for each sensitive attribute (before race upsampling).

		Logistic Regression	Decision Tree	Support Vector Machine	Random Forest	XGBoost	LightGBM
Statistically Significant Precision Score Differences	Sex	No	No	No	No	No	No
	Race	No	No	No	No	No	No
	Device	No	Yes	No	No	Yes	No
	Dominant Hand	No	No	No	No	No	No

Supplementary Table 9: Binary indicator of whether a statistically significance difference in sensitivity is observed using a t-test between groups for each sensitive attribute (before race upsampling).

		Logistic Regression	Decision Tree	Support Vector Machine	Random Forest	XGBoost	LightGBM

**Statistically Significant Sensitivity Score Differences**	**Sex**	No	No	No	No	No	No
	**Race**	Yes	No	Yes	No	No	No
	**Device**	Yes	No	Yes	Yes	No	No
	**Dominant Hand**	No	No	No	No	No	No

Supplementary Table 10: Binary indicator of whether a statistically significance difference in specificity is observed using a t-test between groups for each sensitive attribute (before race upsampling).

		**Logistic Regression**	**Decision Tree**	**Support Vector Machine**	**Random Forest**	**XGBoost**	**LightGBM**
**Statistically Significant Specificity Score Differences**	**Sex**	No	No	No	No	No	No
	**Race**	Yes	No	Yes	No	No	No
	**Device**	Yes	No	Yes	Yes	No	No
	**Dominant Hand**	No	No	No	No	No	No

Supplementary Table 11: Bias metrics of the remaining models based on the sex, race, device type and dominant hand from the cross-validation with bootstrap sampling after race upsampling. Disparate Impact and Equal Opportunity are reported as ratios whereas Equalized Odds is presented as difference between unprivileged and privileged groups.

**Sex**			
	**Disparate Impact**	**Equal Opportunity**	**Equalized Odds**
**Logistic Regression**	0.99 (0.55)	0.99 (0.11)	0.25 (0.17)
**Decision Tree**	1.18 (0.59)	1.02 (0.16)	0.30 (0.23)
**Support Vector Machine**	0.99 (0.57)	1.01 (0.04)	0.22 (0.12)
**Race**			
**Logistic Regression**	1.44 (1.13)	1.17 (0.08)	0.20 (0.05)
**Decision Tree**	1.22 (0.87)	0.99 (0.07)	0.13 (0.05)
**Support Vector Machine**	1.59 (1.28)	1.22 (0.10)	0.21 (0.03)
**Device**			
**Logistic Regression**	0.42 (0.03)	0.90 (0.07)	0.71 (0.27)
**Decision Tree**	0.60 (0.15)	0.99 (0.13)	0.24 (0.15)

**Support Vector Machine**	0.38 (0.04)	0.87 (0.04)	0.74 (0.24)
**Dominant Hand**			
**Logistic Regression**	0.55 (0.18)	0.95 (0.10)	0.15 (0.05)
**Decision Tree**	0.57 (0.22)	1.06 (0.04)	0.11 (0.04)
**Support Vector Machine**	0.57 (0.14)	0.98 (0.12)	0.16 (0.05)

Supplementary Table 12: Bias metrics of the models based on the sex, race, device type and dominant hand from the cross-validation with bootstrap sampling before race upsampling. Disparate Impact and Equal Opportunity are reported as ratios whereas Equalized Odds is presented as difference between unprivileged and privileged groups.

**Sex**			
	**Disparate Impact**	**Equal Opportunity**	**Equalized Odds**
**Logistic Regression**	1.42 (0.48)	1.03 (0.14)	0.34 (0.19)
**Decision Tree**	1.39 (0.68)	0.91 (0.32)	0.46 (0.13)
**Support Vector Machine**	1.21 (0.45)	0.96 (0.20)	0.29 (0.12)
**Random Forest**	1.51 (0.39)	1.03 (0.08)	0.34 (0.14)
**XGBoost**	1.37 (0.48)	1.06 (0.09)	0.34 (0.13)
**LightGBM**	1.35 (0.42)	1.04 (0.09)	0.27 (0.08)
**Race**			
**Logistic Regression**	3.55 (0.76)	1.41 (0.25)	0.57 (0.28)
**Decision Tree**	2.29 (0.51)	1.09 (0.19)	0.24 (0.06)
**Support Vector Machine**	3.83 (1.45)	1.46 (0.33)	0.57 (0.29)
**Random Forest**	2.98 (0.87)	1.06 (0.07)	0.34 (0.29)
**XGBoost**	1.93 (0.68)	0.95 (0.09)	0.47 (0.29)
**LightGBM**	2.80 (0.48)	1.10 (0.14)	0.40 (0.29)
**Device**			
**Logistic Regression**	**0.39 (0.09)**	**0.81 (0.13)**	**0.52 (0.22)**
**Decision Tree**	**0.73 (0.39)**	**1.31 (0.83)**	**0.33 (0.13)**
**Support Vector Machine**	**0.38 (0.06)**	**0.81 (0.11)**	**0.49 (0.26)**
**Random Forest**	**0.45 (0.05)**	**0.91 (0.06)**	**0.26 (0.12)**

**XGBoost**	**0.58 (0.18)**	**0.93 (0.07)**	**0.23 (0.11)**
**LightGBM**	**0.47 (0.03)**	**0.91 (0.07)**	**0.24 (0.08)**
**Dominant Hand**			
**Logistic Regression**	**0.42 (0.18)**	**1.01 (0.05)**	**0.24 (0.03)**
**Decision Tree**	**0.54 (0.20)**	**0.92 (0.22)**	**0.27 (0.07)**
**Support Vector Machine**	**0.48 (0.21)**	**1.00 (0.05)**	**0.21 (0.04)**
**Random Forest**	**0.49 (0.22)**	**0.98 (0.09)**	**0.17 (0.04)**
**XGBoost**	**0.73 (0.18)**	**1.02 (0.15)**	**0.19 (0.06)**
**LightGBM**	**0.55 (0.16)**	**0.98 (0.10)**	**0.19 (0.05)**

Supplementary Table 13: Binary indicator of whether a statistically significant difference in F1-score is observed using a t-test between groups for each sensitive attribute (*before* race upsampling) when excluding demographic features in the training phase.

		**Logistic Regression**	**Decision Tree**	**Support Vector Machine**	**Random Forest**	**XGBoost**	**LightGBM**
**Statistically Significant F1 Score Differences**	**Sex**	No	No	No	No	No	Yes
	**Race**	No	No	No	No	No	Yes
	**Device**	No	No	Yes	Yes	Yes	Yes
	**Dominant Hand**	No	No	Yes	No	No	No

Supplementary Table 14: Bias metrics of the models based on the sex from the cross-validation with bootstrap sampling *before* race upsampling when excluding demographic features in the training phase. Disparate Impact and Equal Opportunity are reported as ratios whereas Equalized Odds is presented as difference between unprivileged and privileged groups.

**Sex**			
	**Disparate Impact**	**Equal Opportunity**	**Equalized Odds**
**Logistic Regression**	1.49 (0.71)	0.98 (0.33)	0.53 (0.28)
**Decision Tree**	1.32 (0.33)	0.93 (0.10)	0.34 (0.15)
**Support Vector Machine**	1.38 (0.45)	1.00 (0.22)	0.37 (0.15)
**Random Forest**	1.42 (0.45)	1.01 (0.14)	0.31 (0.19)

**XGBoost**	1.17 (0.34)	0.94 (0.09)	0.33 (0.13)
**LightGBM**	1.34 (0.15)	0.89 (0.13)	0.39 (0.17)

Supplementary Table 15: Bias metrics of the models based on the race from the cross-validation with bootstrap sampling *before* race upsampling when excluding demographic features in the training phase. Disparate Impact and Equal Opportunity are reported as ratios whereas Equalized Odds is presented as difference between unprivileged and privileged groups..

**Race**			
	**Disparate Impact**	**Equal Opportunity**	**Equalized Odds**
**Logistic Regression**	1.92 (0.34)	0.96 (0.07)	0.35 (0.18)
**Decision Tree**	2.22 (0.94)	1.02 (0.29	0.29 (0.08)
**Support Vector Machine**	1.86 (0.43)	0.97 (0.07)	0.41 (0.18)
**Random Forest**	2.43 (0.75)	0.99 (0.13)	0.20 (0.10)
**XGBoost**	1.82 (0.55)	1.00 (0.21)	0.42 (0.28)
**LightGBM**	2.19 (0.37)	1.07 (0.17)	0.37 (0.21)

Supplementary Table 16: Bias metrics of the models based on the device type from the cross-validation with bootstrap sampling *before* race upsampling when excluding demographic features in the training phase. Disparate Impact and Equal Opportunity are reported as ratios whereas Equalized Odds is presented as difference between unprivileged and privileged groups.

**Device**			
	**Disparate Impact**	**Equal Opportunity**	**Equalized Odds**
**Logistic Regression**	0.58 (0.08)	1.05 (0.24)	0.37 (0.20)
**Decision Tree**	0.59 (0.17)	1.01 (0.25)	0.29 (0.08)
**Support Vector Machine**	0.55 (0.10)	0.92 (0.12)	0.33 (0.12)
**Random Forest**	0.47 (0.05)	0.95 (0.08)	0.26 (0.13)
**XGBoost**	0.59 (0.17)	0.94 (0.10)	0.24 (0.10)
**LightGBM**	0.56 (0.07)	1.01 (0.09)	0.21 (0.07)

Supplementary Table 17: Bias metrics of the models based on the dominant hand from the cross-validation with bootstrap sampling *before* race upsampling when excluding demographic features in the training phase. Disparate Impact and Equal Opportunity are reported as ratios whereas Equalized Odds is presented as difference between unprivileged and privileged groups.

Dominant Hand			
	**Disparate Impact**	**Equal Opportunity**	**Equalized Odds**
**Logistic Regression**	0.50 (0.23)	0.76 (0.41)	0.37 (0.26)
**Decision Tree**	0.61 (0.25)	1.08 (0.19)	0.26 (0.03)
**Support Vector Machine**	0.62 (0.16)	1.01 (0.08)	0.26 (0.13)
**Random Forest**	0.53 (0.20)	1.02 (0.06)	0.15 (0.05)
**XGBoost**	0.70 (0.16)	1.00 (0.10)	0.18 (0.06)
**LightGBM**	0.62 (0.20)	0.98 (0.17)	0.24 (0.07)

Supplementary Table 18: Model performance *before* race upsampling on each group based on sex when excluding demographic features in the training phase.

	**F1 Score**		**Sensitivity**		**Specificity**		**Precision**		**AUROC**	
**Sex**	**Male**	**Female**	**Male**	**Female**	**Male**	**Female**	**Male**	**Female**	**Male**	**Female**
**Logistic Regression**	0.79 (0.04)	0.65 (0.22)	0.82 (0.10)	0.79 (0.29)	0.82 (0.09)	0.52 (0.45)	0.80 (0.08)	0.70 (0.26)	0.89 (0.03)	0.71 (0.31)
**Decision Tree**	0.82 (0.08)	0.71 (0.20)	0.86 (0.12)	0.80 (0.19)	0.81 (0.09)	0.58 (0.26)	0.80 (0.09)	0.70 (0.25)	0.87 (0.09)	0.71 (0.17)
**Support Vector Machine**	0.77 (0.02)	0.69 (0.17)	0.82 (0.13)	0.82 (0.23)	0.78 (0.14)	0.59 (0.34)	0.77 (0.11)	0.70 (0.27)	0.90 (0.04)	0.74 (0.27)
**Random Forest**	0.92 (0.04)	0.83 (0.20)	0.95 (0.05)	0.95 (0.11)	0.90 (0.06)	0.73 (0.29)	0.90 (0.06)	0.79 (0.25)	0.97 (0.03)	0.87 (0.12)
**XGBoost**	0.81 (0.12)	0.74 (0.11)	0.92 (0.09)	0.86 (0.12)	0.68 (0.35)	0.67 (0.20)	0.76 (0.20)	0.70 (0.21)	0.95 (0.04)	0.80 (0.15)
**LightGBM**	0.87 (0.05)	0.68 (0.13)	0.93 (0.06)	0.83 (0.16)	0.84 (0.13)	0.53 (0.30)	0.84 (0.12)	0.65 (0.22)	0.96 (0.03)	0.85 (0.16)

Supplementary Table 19: Model performance *before* race upsampling on each group based on race when excluding demographic features in the training phase.

	**F1 Score**		**Sensitivity**		**Specificity**		**Precision**		**AUROC**	
**Race**	**White**	**Non-White**	**White**	**Non-White**	**White**	**Non-White**	**White**	**Non-White**	**White**	**Non-White**
**Logistic Regression**	0.70 (0.12)	0.85 (0.08)	0.87 (0.11)	0.82 (0.13)	0.79 (0.09)	0.43 (0.39)	0.64 (0.18)	0.91 (0.08)	0.92 (0.01)	0.84 (0.12)
**Decision Tree**	0.69 (0.10)	0.83 (0.16)	0.82 (0.11)	0.81 (0.21)	0.79 (0.09)	0.51 (0.31)	0.64 (0.16)	0.88 (0.12)	0.87 (0.08)	0.59 (0.10)

**Support Vector Machine**	0.66 (0.12)	0.84 (0.09)	0.84 (0.12)	0.80 (0.14)	0.76 (0.15)	0.42 (0.39)	0.62 (0.21)	0.91 (0.08)	0.92 (0.02)	0.87 (0.13)
**Random Forest**	0.83 (0.17)	0.94 (0.04)	0.97 (0.06)	0.95 (0.07)	0.88 (0.08)	0.61 (0.36)	0.77 (0.21)	0.95 (0.07)	0.96 (0.02)	0.95 (0.04)
**XGBoost**	0.69 (0.21)	0.91 (0.06)	0.91 (0.10)	0.89 (0.09)	0.70 (0.30)	0.52 (0.44)	0.63 (0.28)	0.94 (0.08)	0.93 (0.03)	0.95 (0.05)
**LightGBM**	0.72 (0.14)	0.92 (0.05)	0.88 (0.07)	0.93 (0.08)	0.81 (0.13)	0.52 (0.44)	0.67 (0.22)	0.94 (0.08)	0.92 (0.05)	0.99 (0.01)

Supplementary Table 20: Model performance *before* race upsampling on each group based on device type when excluding demographic features in the training phase.

	**F1 Score**		**Sensitivity**		**Specificity**		**Precision**		**AUROC**	
**Device**	**Mac**	**Windows**	**Mac**	**Windows**	**Mac**	**Windows**	**Mac**	**Windows**	**Mac**	**Windows**
**Logistic Regression**	0.86 (0.11)	0.75 (0.05)	0.82 (0.12)	0.83 (0.14)	0.33 (0.41)	0.78 (0.10)	0.92 (0.12)	0.72 (0.08)	0.49 (0.17)	0.88 (0.05)
**Decision Tree**	0.89 (0.12)	0.75 (0.06)	0.87 (0.19)	0.82 (0.08)	0.43 (0.39)	0.79 (0.11)	0.96 (0.05)	0.71 (0.10)	0.91 (0.13)	0.84 (0.09)
**Support Vector Machine**	0.90 (0.06)	0.71 (0.04)	0.88 (0.10)	0.80 (0.16)	0.33 (0.42)	0.75 (0.14)	0.94 (0.09)	0.69 (0.10)	0.66 (0.26)	0.89 (0.04)
**Random Forest**	0.97 (0.03)	0.88 (0.06)	0.98 (0.04)	0.93 (0.07)	0.43 (0.39)	0.89 (0.09)	0.96 (0.05)	0.85 (0.10)	0.93 (0.07)	0.96 (0.02)
**XGBoost**	0.94 (0.04)	0.75 (0.12)	0.95 (0.05)	0.89 (0.11)	0.33 (0.42)	0.69 (0.29)	0.94 (0.09)	0.71 (0.20)	0.95 (0.04)	0.94 (0.04)
**LightGBM**	0.92 (0.04)	0.81 (0.08)	0.90 (0.09)	0.90 (0.06)	0.43 (0.39)	0.80 (0.14)	0.96 (0.05)	0.76 (0.14)	0.87 (0.02)	0.94 (0.04)

Supplementary Table 21: Model performance *before* race upsampling on each group based on dominant hand when excluding demographic features in the training phase.

	**F1 Score**		**Sensitivity**		**Specificity**		**Precision**		**AUROC**	
**Dominant Hand**	**Right**	**Left**	**Right**	**Left**	**Right**	**Left**	**Right**	**Left**	**Right**	**Left**
**Logistic Regression**	0.80 (0.03)	0.54 (0.29)	0.83 (0.09)	0.61 (0.34)	0.71 (0.16)	0.88 (0.06)	0.80 (0.09)	0.55 (0.31)	0.84 (0.06)	0.89 (0.03)
**Decision Tree**	0.79 (0.06)	0.73 (0.19)	0.80 (0.10)	0.85 (0.18)	0.74 (0.13)	0.85 (0.09)	0.80 (0.08)	0.68 (0.22)	0.82 (0.10)	0.84 (0.07)
**Support Vector Machine**	0.78 (0.03)	0.60 (0.13)	0.81 (0.13)	0.80 (0.15)	0.70 (0.22)	0.82 (0.09)	0.79 (0.12)	0.57 (0.22)	0.86 (0.05)	0.92 (0.03)
**Random Forest**	0.91 (0.03)	0.86 (0.16)	0.95 (0.04)	0.96 (0.05)	0.84 (0.08)	0.93 (0.10)	0.88 (0.06)	0.83 (0.23)	0.96 (0.02)	0.96 (0.03)
**XGBoost**	0.82 (0.07)	0.64 (0.30)	0.90 (0.06)	0.89 (0.10)	0.66 (0.28)	0.72 (0.35)	0.79 (0.14)	0.63 (0.38)	0.93 (0.04)	0.93 (0.06)
**LightGBM**	0.85 (0.04)	0.67 (0.20)	0.90 (0.06)	0.87 (0.12)	0.76 (0.17)	0.84 (0.12)	0.83 (0.11)	0.64 (0.29)	0.94 (0.03)	0.94 (0.05)

Supplementary Table 22: Binary indicator of whether a statistically significant difference in F1-score is observed using a t-test between groups for each sensitive attribute (*after* race upsampling) when excluding demographic features in the training phase.

		Logistic Regression	Decision Tree	Support Vector Machine	Random Forest	XGBoost	LightGBM
Statistically Significant F1 Score Differences	Sex	No	No	No	No	No	No
	Race	No	No	No	No	No	No
	Device	No	No	No	Yes	No	No
	Dominant Hand	No	No	No	No	No	No

Supplementary Table 23: Bias metrics of the models based on the sex from the cross-validation with bootstrap sampling *after* race upsampling when excluding demographic features in the training phase. Disparate Impact and Equal Opportunity are reported as ratios whereas Equalized Odds is presented as difference between unprivileged and privileged groups.

Sex			
	Disparate Impact	Equal Opportunity	Equalized Odds
Logistic Regression	0.97 (0.50)	0.98 (0.11)	0.27 (0.15)
Decision Tree	1.02 (0.54)	1.06 (0.20)	0.24 (0.18)
Support Vector Machine	0.88 (0.39)	0.94 (0.14)	0.21 (0.05)
Random Forest	1.03 (0.54)	1.00 (0.04)	0.20 (0.22)
XGBoost	0.97 (0.52)	0.97 (0.14)	0.31 (0.21)
LightGBM	1.06 (0.52)	1.00 (0.04)	0.20 (0.22)

Supplementary Table 24: Bias metrics of the models based on the race from the cross-validation with bootstrap sampling *after* race upsampling when excluding demographic features in the training phase. Disparate Impact and Equal Opportunity are reported as ratios whereas Equalized Odds is presented as difference between unprivileged and privileged groups.

Race			
	Disparate Impact	Equal Opportunity	Equalized Odds
Logistic Regression	1.32 (1.06)	1.07 (0.09)	0.20 (0.07)
Decision Tree	1.44 (1.14)	1.14 (0.21)	0.15 (0.10)
Support Vector Machine	1.39 (1.16)	1.09 (0.11)	0.20 (0.07)
Random Forest	1.47 (1.20)	1.01 (0.01)	0.07 (0.04)

**XGBoost**	1.29 (1.22)	1.07 (0.10)	0.23 (0.28)
**LightGBM**	1.37 (1.32)	1.05 (0.04)	0.19 (0.26)

Supplementary Table 25: Bias metrics of the models based on the device type from the cross-validation with bootstrap sampling *after* race upsampling when excluding demographic features in the training phase. Disparate Impact and Equal Opportunity are reported as ratios whereas Equalized Odds is presented as difference between unprivileged and privileged groups.

Device			
	**Disparate Impact**	**Equal Opportunity**	**Equalized Odds**
**Logistic Regression**	0.47 (0.08)	0.97 (0.04)	0.60 (0.36)
**Decision Tree**	0.63 (0.07)	1.16 (0.27)	0.23 (0.15)
**Support Vector Machine**	0.47 (0.06)	0.98 (0.03)	0.63 (0.31)
**Random Forest**	0.55 (0.12)	0.98 (0.03)	0.15 (0.16)
**XGBoost**	0.63 (0.12)	1.00 (0.02)	0.11 (0.05)
**LightGBM**	0.57 (0.08)	0.98 (0.03)	0.17 (0.22)

Supplementary Table 26: Bias metrics of the models based on the dominant hand from the cross-validation with bootstrap sampling *after* race upsampling when excluding demographic features in the training phase. Disparate Impact and Equal Opportunity are reported as ratios whereas Equalized Odds is presented as difference between unprivileged and privileged groups.

Dominant Hand			
	**Disparate Impact**	**Equal Opportunity**	**Equalized Odds**
**Logistic Regression**	0.55 (0.17)	0.98 (0.10)	0.17 (0.03)
**Decision Tree**	0.49 (0.16)	1.00 (0.17)	0.18 (0.06)
**Support Vector Machine**	0.56 (0.16)	0.96 (0.10)	0.18 (0.03)
**Random Forest**	0.55 (0.23)	1.01 (0.03)	0.07 (0.04)
**XGBoost**	0.66 (0.25)	0.96 (0.09)	0.13 (0.07)
**LightGBM**	0.59 (0.18)	0.97 (0.05)	0.09 (0.07)

Supplementary Table 27: Model performance *after* race upsampling on each group based on sex when excluding demographic features in the training phase.

	F1 Score		Sensitivity		Specificity		Precision		AUROC	
**Sex**	**Male**	**Female**	**Male**	**Female**	**Male**	**Female**	**Male**	**Female**	**Male**	**Female**

**Logistic Regression**	0.86 (0.02)	0.83 (0.14)	0.90 (0.05)	0.87 (0.11)	0.84 (0.05)	0.76 (0.25)	0.84 (0.04)	0.81 (0.18)	0.95 (0.02)	0.80 (0.21)
**Decision Tree**	0.87 (0.06)	0.88 (0.13)	0.84 (0.09)	0.88 (0.17)	0.93 (0.04)	0.80 (0.26)	0.91 (0.05)	0.90 (0.13)	0.91 (0.06)	0.83 (0.19)
**Support Vector Machine**	0.85 (0.02)	0.82 (0.16)	0.89 (0.05)	0.84 (0.14)	0.82 (0.07)	0.80 (0.16)	0.82 (0.04)	0.82 (0.19)	0.94 (0.02)	0.84 (0.15)
**Random Forest**	0.96 (0.02)	0.93 (0.07)	0.98 (0.02)	0.98 (0.05)	0.94 (0.02)	0.78 (0.26)	0.94 (0.02)	0.89 (0.09)	0.99 (0.01)	0.94 (0.08)
**XGBoost**	0.89 (0.09)	0.84 (0.15)	0.93 (0.04)	0.90 (0.15)	0.83 (0.22)	0.72 (0.27)	0.86 (0.14)	0.81 (0.17)	0.97 (0.03)	0.86 (0.16)
**LightGBM**	0.93 (0.08)	0.88 (0.12)	0.98 (0.03)	0.97 (0.05)	0.88 (0.17)	0.72 (0.26)	0.90 (0.13)	0.82 (0.18)	0.99 (0.01)	0.90 (0.13)

Supplementary Table 28: Model performance *after* race upsampling on each group based on race when excluding demographic features in the training phase.

	**F1 Score**		**Sensitivity**		**Specificity**		**Precision**		**AUROC**	
**Race**	**White**	**Non-White**	**White**	**Non-White**	**White**	**Non-White**	**White**	**Non-White**	**White**	**Non-White**
**Logistic Regression**	0.80 (0.11)	0.86 (0.08)	0.84 (0.07)	0.89 (0.06)	0.47 (0.39)	0.54 (0.44)	0.80 (0.18)	0.86 (0.15)	0.89 (0.03)	0.98 (0.01)
**Decision Tree**	0.83 (0.13)	0.92 (0.05)	0.82 (0.14)	0.90 (0.11)	0.53 (0.44)	0.59 (0.48)	0.86 (0.15)	0.96 (0.05)	0.81 (0.08)	0.99 (0.01)
**Support Vector Machine**	0.80 (0.11)	0.86 (0.07)	0.83 (0.08)	0.89 (0.06)	0.46 (0.39)	0.53 (0.43)	0.79 (0.19)	0.85 (0.14)	0.90 (0.02)	0.97 (0.01)
**Random Forest**	0.93 (0.06)	0.96 (0.02)	0.97 (0.03)	0.98 (0.02)	0.54 (0.44)	0.58 (0.47)	0.89 (0.09)	0.95 (0.05)	0.98 (0.01)	1.00 (0.00)
**XGBoost**	0.81 (0.19)	0.95 (0.01)	0.91 (0.10)	0.96 (0.05)	0.39 (0.41)	0.58 (0.47)	0.77 (0.24)	0.95 (0.04)	0.91 (0.06)	1.00 (0.00)
**LightGBM**	0.85 (0.15)	1.00 (0.01)	0.95 (0.04)	0.99 (0.02)	0.42 (0.40)	0.60 (0.49)	0.80 (0.21)	1.00 (0.00)	0.95 (0.02)	1.00 (0.00)

Supplementary Table 29: Model performance *after* race upsampling on each group based on device type when excluding demographic features in the training phase.

	**F1 Score**		**Sensitivity**		**Specificity**		**Precision**		**AUROC**	
**Device**	**Mac**	**Windows**	**Mac**	**Windows**	**Mac**	**Windows**	**Mac**	**Windows**	**Mac**	**Windows**
**Logistic Regression**	0.84 (0.09)	0.85 (0.06)	0.91 (0.07)	0.88 (0.05)	0.40 (0.49)	0.88 (0.07)	0.81 (0.17)	0.82 (0.09)	0.80 (0.18)	0.94 (0.04)
**Decision Tree**	0.86 (0.14)	0.87 (0.08)	0.79 (0.19)	0.86 (0.09)	0.90 (0.20)	0.92 (0.07)	0.97 (0.07)	0.89 (0.10)	0.84 (0.17)	0.90 (0.10)
**Support Vector Machine**	0.82 (0.09)	0.83 (0.07)	0.90 (0.09)	0.88 (0.07)	0.30 (0.40)	0.87 (0.08)	0.79 (0.17)	0.81 (0.10)	0.76 (0.15)	0.94 (0.03)
**Random Forest**	0.99 (0.02)	0.93 (0.04)	0.99 (0.02)	0.97 (0.03)	0.90 (0.21)	0.93 (0.04)	0.98 (0.04)	0.90 (0.05)	0.99 (0.02)	0.99 (0.01)

**XGBoost**	0.96 (0.04)	0.85 (0.12)	0.93 (0.05)	0.93 (0.05)	0.90 (0.19)	0.83 (0.18)	0.98 (0.04)	0.81 (0.17)	0.96 (0.06)	0.96 (0.03)
**LightGBM**	0.97 (0.04)	0.89 (0.11)	0.99 (0.03)	0.97 (0.03)	0.80 (0.40)	0.87 (0.15)	0.97 (0.07)	0.85 (0.17)	0.99 (0.02)	0.98 (0.02)

Supplementary Table 30: Model performance *after* race upsampling on each group based on dominant hand when excluding demographic features in the training phase.

	**F1 Score**		**Sensitivity**		**Specificity**		**Precision**		**AUROC**	
**Dominant Hand**	**Right**	**Left**	**Right**	**Left**	**Right**	**Left**	**Right**	**Left**	**Right**	**Left**
**Logistic Regression**	0.87 (0.02)	0.80 (0.12)	0.90 (0.04)	0.88 (0.10)	0.83 (0.07)	0.91 (0.08)	0.85 (0.05)	0.78 (0.20)	0.93 (0.04)	0.95 (0.04)
**Decision Tree**	0.89 (0.07)	0.88 (0.11)	0.88 (0.10)	0.86 (0.11)	0.90 (0.08)	0.96 (0.06)	0.91 (0.06)	0.91 (0.13)	0.89 (0.09)	0.88 (0.07)
**Support Vector Machine**	0.86 (0.03)	0.76 (0.15)	0.88 (0.05)	0.85 (0.12)	0.82 (0.10)	0.89 (0.09)	0.84 (0.07)	0.75 (0.23)	0.93 (0.04)	0.95 (0.05)
**Random Forest**	0.95 (0.02)	0.91 (0.08)	0.97 (0.02)	0.98 (0.04)	0.92 (0.05)	0.96 (0.04)	0.93 (0.03)	0.87 (0.14)	0.99 (0.01)	0.98 (0.02)
**XGBoost**	0.90 (0.07)	0.77 (0.27)	0.94 (0.04)	0.91 (0.11)	0.83 (0.16)	0.84 (0.25)	0.87 (0.10)	0.76 (0.32)	0.97 (0.02)	0.98 (0.03)
**LightGBM**	0.93 (0.07)	0.82 (0.22)	0.98 (0.02)	0.95 (0.06)	0.86 (0.17)	0.90 (0.13)	0.90 (0.12)	0.79 (0.28)	0.98 (0.01)	0.98 (0.03)

Supplementary Table 31: List of all features used in the models.

**Category**	**Sub Category**	**Feature Name**	**Feature Description**	**Unit**
Mouse Movement	Straight Line	Mean deviation straight line	Mean deviation from centerline when tracing straight line (fraction of screen height)	#
		Maximum deviation straight line	Maximum deviation from centerline when tracing straight line (percentage of screen height)	#
		Average absolute deviation straight line	Average of absolute values of deviation from centerline when tracing straight line (percentage of screen height)	#
		Net deviation straight line	Net accumulated deviation from centerline when tracing straight line (percentage of screen height)	#

		Total deviation straight line	Total accumulated deviation from centerline when tracing straight line (percentage of screen height)	#
		Maximum pixel deviation straight line	Maximum deviation from centerline when tracing straight line without regard to window height (pixels)	#
		Time to trace straight line	Amount of time taken to trace straight line	Milliseconds
		Time to trace straight line normalized	Amount of time taken to trace straight line with respect to window width	Milliseconds
		Percent points inside straight line	Percentage of points traced inside straight line	%
		Total points inside straight line	Number of points traced inside straight line with no regard to time taken	#
	Sine Wave	Time to trace sine wave	Amount of time taken to trace sine wave	Milliseconds
		Time to trace sine wave normalized	Amount of time taken to trace sine wave with respect to window width	Milliseconds
		Percent points inside sine wave	Percentage of points traced inside sine wave	%
	Sine Wave	Total points inside sine wave	Number of points traced inside sine wave with no regard to time taken	#
	Spiral	Time to trace spiral	Amount of time taken to trace spiral	Milliseconds
		Time to trace spiral normalized	Amount of time taken to trace spiral with respect to window width	Milliseconds
		Percent points inside spiral	Percentage of points traced inside spiral	%
		Total points inside spiral	Percentage of points traced inside spiral with no regard to time taken	%
	All Test	Average tracing time all tasks	Average time taken to complete all line-tracing tasks.	Milliseconds

		Normalized tracing time all tasks	Average time taken to complete all line-tracing tasks normalized by the width of the application window to account for screen size differences.	Milliseconds per pixel
		Average points inside all lines	Average number of mouse trace points falling within the line boundaries, regardless of the time taken.	#
		Ratio of sine to straight points	Ratio of percentage of points traced inside the sine wave path to those inside a straight-line path.	#
		Ratio of spiral to straight points	Ratio of percentage of points traced within the spiral path to those within a straight line	#
		Ratio of sine to spiral points	Ratio of percentage of points inside the sine wave path to those inside the spiral path	#
Keyboard Button Press	Constant Key	False presses constant key	False presses when prompted with a constant key	#
		Correct presses constant key	Number of correctly pressed keys when prompted with a constant key	#
		Average response time constant key	Average response time when prompted with a constant key	Milliseconds
		Total response time constant key	Sum of response times when prompted with a constant key	Milliseconds
	Constant Key	False press ratio constant key	Ratio of false key presses to the total number when prompted with a constant key	#
		Correct press rate constant key	Ratio of correct key presses to the average response time when prompted with a constant key.	#

	Semi Random (Fixed Between Two Random Letters)	False presses semi-random key	False presses when prompted with a semi-random key	#
		Correct presses semi-random key	Number of correctly pressed keys when prompted with a semi-random key	#
		Average response time semi-random key	Average response time when prompted with a semi-random key	Milliseconds
		Total response time semi-random key	Sum of response times when prompted with a semi-random	Milliseconds
		False press ratio semi-random key	Ratio of false key presses to the total number when prompted with a semi-random key	#
		Correct press rate semi-random key	Ratio of correct key presses to the average response time when prompted with semi-random keys	#
	Random Key	False presses random key	False presses when prompted with a random key	#
		Correct presses random key	Number of correctly pressed keys when prompted with a random key	#
		Average response time random key	Average response time when prompted with a random key	Milliseconds
		Total response time random key	Sum of response times when prompted with a random	Milliseconds
		Correct press rate random key	Number of correctly pressed keys when prompted with a random key with respect to average response time	#
		False press ratio random key	Ratio of false key presses to the total number when prompted with a random key	#
	All Test	Total false presses all tests	Total false presses from all tests	#

	All Test	Average false presses all tests	Average false presses from all tests	#
		Average false press ratio all tests	Average ratio of false key presses to total key presses	#
		Std of false press ratio all tests	Standard deviation of ratio of false key presses to total key presses	#
	Dominant Hand	Total P key time dominant hand	Total Time from Dominant Hands for pressing p	Milliseconds
		Average P key time dominant hand	Average Time from Dominant Hands for pressing P	Milliseconds
		Total P key time non-dominant hand	Total Time from Non-Dominant Hands for pressing P	Milliseconds
		Average P key time non-dominant hand	Average Time from Non-Dominant Hands for pressing P	Milliseconds
		Ratio of P key time dominant to non-dominant	Ratio of Average Time from Dominant Hand and Non-Dominant Hand for pressing P	#
		Total Q key time dominant hand	Total Time from Dominant Hands for pressing Q	Milliseconds
		Average Q key time dominant hand	Average Time from Dominant Hands for pressing Q	Milliseconds
		Total Q key time non-dominant hand	Total Time from Non-Dominant Hands for pressing Q	Milliseconds
		Average Q key time non-dominant hand	Average Time from Non-Dominant Hands for pressing Q	Milliseconds
		Ratio of Q key time dominant to non-dominant	Ratio of Average Time from Dominant Hand and Non-Dominant Hand for pressing Q	#
Mouse-Keyboard Combined		Straight trace time × false presses	Product of total time taken to trace straight lines and the number of false key presses when prompted with a constant key	#

		Sine wave trace time × false presses	Product of time spent tracing sine wave patterns and the number of false key presses during constant key prompts	#
		Spiral trace time × false presses	Product of time taken to trace spirals and false key presses when prompted with a constant key	#
Mouse Click	Correct/ Right Clicks	Total correct clicks	Total Right Clicks	#
		Average correct clicks	Mean Right Clicks	#
		Std of correct clicks	Standard Deviation Right Clicks	#
	Wrong Clicks	Total wrong clicks	Total Wrong clicks	#
		Average wrong clicks	Mean Wrong clicks	#
		Std of wrong clicks	Standard Deviation Wrong Clicks	#
	All Test	Total click reaction time	Total Reaction Time for Data collected from Game	Milliseconds
		Average click reaction time	Average Reaction time for Data collected from Game	Milliseconds
		Std of click reaction time	Standard Deviation of Reaction Time for Data collected from Game	Milliseconds
		Range of click reaction time	Difference of Maximum and Minimum Reaction time for Data Collected from Game	Milliseconds
Memory Game		Total correct memory clicks	Correct number of Box Clicks	#
		Total click time all tests	Total Time taken for clicking Box	Milliseconds
		Average click time all tests	Average Time taken for clicking Box	Milliseconds
		Std of click time all tests	Standard Deviation of Time taken for clicking Box	Milliseconds
Demographic Information		Height	Participant Height	#
		Age	Age	#

(We trained a model both with and without these features)		Sex	Sex	[Male, Female]
		Race	Race	String
		Dominant Hand	Dominant Hand	[Left, Right]
		Device Type	Device Type	[Windows, Mac]

Supplementary Table 32: Top 10 features based on dominant hand and device type according to a random forest model without race upsampling.

Right-handed			
**Rank**	**Feature Name**	**Importance**	**Feature Description**
1	Maximum pixel deviation straight line	0.04	Maximum deviation from centerline when tracing straight line without regard to window height (pixels)
2	Time to trace straight line	0.04	Amount of time taken to trace straight line
3	Maximum deviation straight line	0.04	Maximum deviation from centerline when tracing straight line (percentage of screen height)
4	Time to trace sine wave normalized	0.04	Amount of time taken to trace sine wave with respect to window width
5	Total deviation straight line	0.04	Total accumulated deviation from centerline when tracing straight line (percentage of screen height)
6	Time to trace spiral normalized	0.04	Amount of time taken to trace spiral with respect to window width
7	Net deviation straight line	0.04	Net accumulated deviation from centerline when tracing straight line (percentage of screen height)
8	Average tracing time all tasks	0.03	Average time taken to complete all line-tracing tasks.
9	Time to trace straight line normalized	0.03	Amount of time taken to trace straight line with respect to window width
10	Percent points inside straight line	0.03	Percentage of points traced inside straight line
**Left-handed**			
**Rank**	**Feature Name**	**Importance**	**Feature Description**
1	Correct press rate constant key	0.08	Ratio of correct key presses to the average response time when prompted with a constant key.
2	Average absolute deviation straight line	0.07	Average of absolute values of deviation from centerline when tracing straight line (percentage of screen height)
3	Maximum pixel deviation straight line	0.05	Maximum deviation from centerline when tracing straight line without regard to window height (pixels)
4	Average response time constant key	0.05	Average response time when prompted with a constant key

5	Mean deviation straight line	0.05	Mean deviation from centerline when tracing straight line (fraction of screen height)
6	Total response time semi-random key	0.05	Sum of response times when prompted with a semi-random
7	Total deviation straight line	0.04	Total accumulated deviation from centerline when tracing straight line (percentage of screen height)
8	Total Q key time non-dominant hand	0.04	Total time from non-dominant hands for pressing q
9	Time to trace spiral	0.03	Amount of time taken to trace spiral
10	Time to trace spiral normalized	0.03	Amount of time taken to trace spiral with respect to window width
**Windows**			
**Rank**	**Feature Name**	**Importance**	**Feature Description**
1	Time to trace sine wave normalized	0.1	Amount of time taken to trace sine wave with respect to window width
2	Time to trace straight line	0.06	Amount of time taken to trace straight line
3	Average points inside all lines	0.06	Average number of mouse trace points falling within the line boundaries, regardless of the time taken.
4	Time to trace straight line normalized	0.05	Amount of time taken to trace straight line with respect to window width
5	Time to trace spiral	0.05	Amount of time taken to trace spiral
6	Net deviation straight line	0.04	Net accumulated deviation from centerline when tracing straight line (percentage of screen height)
7	Average tracing time all tasks	0.04	Average time taken to complete all line-tracing tasks.
8	Total deviation straight line	0.04	Total accumulated deviation from centerline when tracing straight line (percentage of screen height)
9	Time to trace sine wave	0.04	Amount of time taken to trace sine wave
10	Total points inside spiral	0.03	Percentage of points traced inside spiral with no regard to time taken
**Mac**			
**Rank**	**Feature Name**	**Importance**	**Feature Description**
1	Total click time all tests	0.06	Total time taken for clicking box
2	False press ratio random key	0.05	Ratio of false key presses to the total number when prompted with a random key
3	Maximum pixel deviation straight line	0.05	Maximum deviation from centerline when tracing straight line without regard to window height (pixels)
4	Average false presses all tests	0.05	Average false presses from all tests
5	Total click reaction time	0.04	Total reaction time for data collected from click game
6	Average click time all tests	0.04	Average time taken for clicking box
7	Total points inside spiral	0.03	Percentage of points traced inside spiral with no regard to time taken

8	Ratio of Q key time dominant to non-dominant	0.03	Ratio of average time from dominant hand and non-dominant hand for pressing q
9	Average correct clicks	0.03	Mean right clicks
10	Maximum deviation straight line	0.03	Maximum deviation from centerline when tracing straight line (percentage of screen height)

Supplementary Table 33: Globally top-ranked features according to a random forest model after race upsampling.

Rank	Feature Name	Importance	Feature Description
**1**	Total deviation straight line	0.07	Total accumulated deviation from centerline when tracing straight line (percentage of screen height)
**2**	Average absolute deviation straight line	0.06	Average of absolute values of deviation from centerline when tracing straight line (percentage of screen height)
**3**	Average tracing time all tasks	0.05	Average time taken to complete all line-tracing tasks.
**4**	Net deviation straight line	0.04	Net accumulated deviation from centerline when tracing straight line (percentage of screen height)
**5**	Time to trace straight line	0.03	Amount of time taken to trace straight line
**6**	Mean deviation straight line	0.03	Mean deviation from centerline when tracing straight line (fraction of screen height)
**7**	Time to trace sine wave	0.03	Amount of time taken to trace sine wave
**8**	Percent points inside straight line	0.03	Percentage of points traced inside straight line
**9**	Time to trace spiral	0.03	Amount of time taken to trace spiral
**10**	Device type	0.03	Device used by the participant

Supplementary Table 34: Top-ranked features for right-handed individuals according to a random forest model after race upsampling.

Rank	Feature Name	Importance	Feature Description
**1**	Total deviation straight line	0.07	Total accumulated deviation from centerline when tracing straight line (percentage of screen height)
**2**	Time to trace straight line	0.04	Amount of time taken to trace straight line
**3**	Net deviation straight line	0.04	Net accumulated deviation from centerline when tracing straight line (percentage of screen height)
**4**	Time to trace spiral	0.04	Amount of time taken to trace spiral
**5**	Average tracing time all tasks	0.04	Average time taken to complete all line-tracing tasks.
**6**	Average absolute deviation straight line	0.04	Average of absolute values of deviation from centerline when tracing straight line (percentage of screen height)
**7**	Time to trace sine wave	0.03	Amount of time taken to trace sine wave
**8**	Mean deviation straight line	0.03	Mean deviation from centerline when tracing straight line (fraction of screen height)

**9**	Percent points inside straight line	0.03	Percentage of points traced inside straight line
**10**	Maximum pixel deviation straight line	0.03	Maximum deviation from centerline when tracing straight line without regard to window height (pixels)

Supplementary Table 35: Top-ranked features for Windows devices according to a random forest model after race upsampling.

Rank	Feature Name	Importance	Feature Description
**1**	Total deviation straight line	0.07	Total accumulated deviation from centerline when tracing straight line (percentage of screen height)
**2**	Time to trace straight line	0.06	Amount of time taken to trace straight line
**3**	Average points inside all lines	0.05	Average number of mouse trace points falling within the line boundaries, regardless of the time taken.
**4**	Average tracing time all tasks	0.05	Average time taken to complete all line-tracing tasks.
**5**	Average absolute deviation straight line	0.04	Average of absolute values of deviation from centerline when tracing straight line (percentage of screen height)
**6**	Time to trace sine wave	0.04	Amount of time taken to trace sine wave
**7**	Total points inside spiral	0.04	Percentage of points traced inside spiral with no regard to time taken
**8**	Time to trace spiral	0.04	Amount of time taken to trace spiral
**9**	Time to trace sine wave normalized	0.03	Amount of time taken to trace sine wave with respect to window width
**10**	Time to trace spiral normalized	0.03	Amount of time taken to trace spiral with respect to window width

Supplementary Table 36: Top-ranked features for left-handed individuals according to a random forest model after race upsampling.

Rank	Feature Name	Importance	Feature Description
**1**	Average absolute deviation straight line	0.08	Average of absolute values of deviation from centerline when tracing straight line (percentage of screen height)
**2**	Total deviation straight line	0.06	Total accumulated deviation from centerline when tracing straight line (percentage of screen height)
**3**	Correct press rate constant key	0.05	Ratio of correct key presses to the average response time when prompted with a constant key.
**4**	Total response time constant key	0.04	Sum of response times when prompted with a constant key
**5**	Average Q key time dominant hand	0.04	Average time from dominant hands for pressing q
**6**	Device Windows	0.04	Participant who used Windows device to access the website
**7**	Time to trace spiral normalized	0.03	Amount of time taken to trace spiral with respect to window width
**8**	Percent points inside straight line	0.03	Percentage of points traced inside straight line

**9**	Maximum deviation straight line	0.03	Maximum deviation from centerline when tracing straight line (percentage of screen height)
**10**	Correct press rate random key	0.03	Number of correctly pressed keys when prompted with a random key with respect to average response time

Supplementary Table 37: Top-ranked features for Mac devices according to a random forest model after race upsampling.

Rank	Feature Name	Importance	Feature Description
**1**	Total click time all tests	0.07	Total time taken for clicking box
**2**	Average click time all tests	0.07	Average time taken for clicking box
**3**	Maximum pixel deviation straight line	0.05	Maximum deviation from centerline when tracing straight line without regard to window height (pixels)
**4**	Mean deviation straight line	0.04	Mean deviation from centerline when tracing straight line (fraction of screen height)
**5**	Net deviation straight line	0.04	Net accumulated deviation from centerline when tracing straight line (percentage of screen height)
**6**	False presses semi-random key	0.03	False presses when prompted with a semi-random key
**7**	Percent points inside spiral	0.03	Percentage of points traced inside spiral
**8**	Percent points inside sine wave	0.03	Percentage of points traced inside sine wave
**9**	Correct press rate random key	0.03	Number of correctly pressed keys when prompted with a random key with respect to average response time
**10**	Average P key time non-dominant hand	0.03	Average time from non-dominant hands for pressing p
